# The coast of giants: an anthropometric survey of high schoolers on the Adriatic coast of Croatia

**DOI:** 10.7717/peerj.6598

**Published:** 2019-04-17

**Authors:** Pavel Grasgruber, Stipan Prce, Nikola Stračárová, Eduard Hrazdíra, Jan Cacek, Stevo Popović, Sylva Hřebíčková, Predrag Potpara, Ivan Davidovič, Tomáš Kalina

**Affiliations:** 1 Faculty of Sports Studies, Masaryk University, Brno, Czech Republic; 2 Gimnazija Metković, Metković, Croatia; 3 Faculty for Sport and Physical Education, University of Montenegro, Nikšić, Montenegro; 4 Ekonomska škola, Bar, Montenegro

**Keywords:** Croatia, Dalmatia, Dinaric Alps, Height, Body proportions

## Abstract

The aim of this anthropometric survey was to map regional differences in height and body proportions in eight counties adjacent to the Adriatic coast of Croatia. Body height was measured in 1,803 males and 782 females aged 17–20 years at 66 schools in 23 towns. When corrected for population size in regions, mean male height is 182.6 cm in all eight counties, 182.8 cm in seven counties of Adriatic Croatia, and 183.7 cm in four counties of Dalmatia proper. Regional variation is considerable: from 180.6 cm in the county of Karlovac to 184.1 cm in the county of Split-Dalmacija. The mean height of females is based on more limited data (168.0 cm in seven counties). These results show that young men from Dalmatia are currently the tallest in the world in the age category of 18 years, and the north-to-south gradient of increasing stature on the Adriatic coast largely mirrors that in neighbouring Bosnia and Herzegovina (BiH). The extraordinary values of height in Croatia and BiH can most likely be explained by unique genetic predispositions that are shared by the local populations of the Dinaric Alps.

## Introduction

Our previous study dealing with the stature of young males in Bosnia and Herzegovina (BiH) (Grasgruber et al., 2017) confirmed older reports showing that people from the Dinaric Alps are among of the tallest in the world. Currently, there do not exist any detailed studies attempting to explain this remarkable phenomenon, but our ecological comparisons indicate that extraordinary genetic predispositions of the local population are the most likely answer (Grasgruber et al., 2014). More concretely, physical stature in Europe shows a remarkable relationship with the frequency of Y haplogroup (paternal lineage) I-M170. Already at the dawn of population genetic research, it was suggested that Y haplogroup I-M170 has its roots in the Upper Paleolithic Gravettian culture and reflects the expansion of Epigravettian (late Gravettian) groups from a glacial ‘refugium’ around the Adriatic Sea (Semino et al., 2000). Indeed, the oldest sample of I-M170 documented in Europe belongs to a Gravettian male from Paglicci in southern Italy (∼32,900 calibrated ^14^C years ago) (Fu et al., 2016). At present, I-M170 has two main frequency peaks among the Germanic-speaking nations of Central and Northern Europe, and in the Western Balkans (Rootsi et al., 2004).

In any case, it is obvious that the genetic potential of Western Balkan populations is far from fully expressed due to troubled local economies and a relatively poor quality of their diet, but given the presence of these negative environmental factors, their tallness is even more remarkable. The mean height of young men from Herzegovina aged 17–20 years is 183.4 cm, which is only slightly lower than in the wealthy and well-fed Dutch (183.8 cm), who are officially the tallest in the world (Schönbeck et al., 2012).

Areas with extraordinary height are not limited to Herzegovina and have an analogy in neighbouring Montenegro, and on the Adriatic coast of Croatia (Dalmatia). The mean height of young Montenegrin males aged 17–20 years is 183.4 cm (Popović, 2017), although this value would slightly decrease to 182.9 cm, if we took population size in individual municipalities into account. In another study performed between 2001–2003 in Dalmatia (the towns of Split, Šibenik, Drniš, Sinj, Imotski, Vrgorac and Dubrovnik), Pineau, Delamarche & Bozinovic (2005) reported a mean height of 183.8 cm (*n* = 1,253) in local males aged 17 years (J-C Pineau, pers. comm., 2013). However, the pooled mean of 18-to-19-year old boys in the Croatian capital of Zagreb was only 180.1 cm in 2010 (*n* = 133) (Petranović et al., 2014). In a nationwide study performed between 2006–2008, the mean height of Croatian boys aged 18 years was 180.5 cm (*n* = 358) (Jureša, Musil & Kujundžić Tiljak, 2012).

These data strongly suggest that the tall statures documented in Dalmatia are rather a geographical exception and height decreases rapidly in the northern direction towards mainland Croatia. Indeed, historical data show that Croatian recruits around 1883 were 165.5 cm tall (Komlos, 2007), but mean height on the Adriatic coast ranged from 166 cm in Istria to 171 cm in southernmost Dalmatia (Coon, 1939 p, 591). A large nationwide survey of school children performed in Croatia between 1980–1984 in 36 towns/areas showed that these differences had persisted even after 100 years (Prebeg, 1988). At the age of 18 years, mean height in some coastal and mainland regions differed by 4–5 cm. The shortest high schoolers came from the region of Zagorje north of Zagreb. Because few data from this research have been published, and, to our knowledge, no later anthropometric survey used sufficiently representative regional samples of young males and females from the territory of Croatia, there is a gap of more than 100 years in the study of this problem. At the same time, such detailed regional data would be very important for the purpose of our research because they would allow us to see how the values of height change across multiple countries and ethnicities, geographic conditions, altitude, economic wealth etc., giving us more insight into the determinants of height variation. Therefore, mapping these geographical differences constitutes an intriguing challenge and the Adriatic coast is a particularly interesting territory in this regard.

Geographically, Adriatic Croatia is divided into two halves, which are separated by the mountain ranges of Velebit and Kapela. These mountains are a continuation of the main ridge of the Dinaric Alps in BiH and run through the rocky and sparsely inhabited county of Lika-Senj. They also separate the whole Adriatic coast from mainland Croatia. The region south of this mountain range is traditionally considered as ‘Dalmatia proper’ and consists of four counties (Zadar, Šibenik-Knin, Split-Dalmacija and Dubrovnik-Neretva).

Although Dalmatia has a very pleasant Mediterranean climate, it has suffered from severe deforestation since the Neolithic (Kranjc, 2009), which led to the creation of an unhospitable ‘limestone desert’ covered by grass and bush. The local population has historically relied on pastoralism and at present, inland Dalmatia is characterized by high rates of emigration for economic reasons. Furthermore, during the war events of 1991–1995, ethnic tensions in the counties of Šibenik-Knin, Zadar, Lika-Senj and Karlovac led to the displacement of the local Serbian population. After the end of the war, the areas experienced an influx of Croatian (Catholic) refugees from BiH.

Because of these ongoing population changes, our research is one of the last opportunities to capture the original distribution of anthropological characteristics in this region. We were particularly interested in whether the north-to-south height gradient follows that documented in BiH—from 180.0 cm in Canton Una-Sana to 184.5 cm in the region of Trebinje. Therefore, we decided to include the whole area of coastal Croatia adjacent to the border of BiH.

## Methods

The research on the Adriatic coast of Croatia is a part of a larger anthropological project started in collaboration with the University of Montenegro in 2015. The aim of this project is to provide detailed mapping of body height and some other anthropological characteristics on the territory of the Dinaric Alps, which would enable better understanding of this intriguing phenomenon. At present, this project already covers Bosnia, Herzegovina, Croatia, Montenegro and Kosovo. In addition, preliminary measurements of university students were performed in Albania during the year 2017. The survey in Montenegro will be repeated in 2019.

The primary objective of the present survey was to measure body height and some body proportions (sitting height, arm span) on the territory of Adriatic Croatia, as defined by the Croatian Bureau of Statistics (Narodne Novine, 2012). The results will serve both for the purpose of the anthropological research in the Dinaric Alps and for our present research aimed at the identification of world regions with the highest sports potential because height and body proportions are one of the most important predictors of sports performance (Ackland, Elliott & Bloomfield, 2009).

The survey and all the planned procedures were approved by the Ethics Committee of the Faculty of Sports Studies, Masaryk University Brno, under the reference number EKV-2016-094. The Ethics Committee assessed the project and found no contradictions with applicable rules, regulations and international guidelines for biomedical research involving human participants. The research was subsequently approved by the Ministry of Education in Croatia, but the final approval depended on individual school directors and their agreement with the students‘ parents.

### Target population

**Figure 1:**
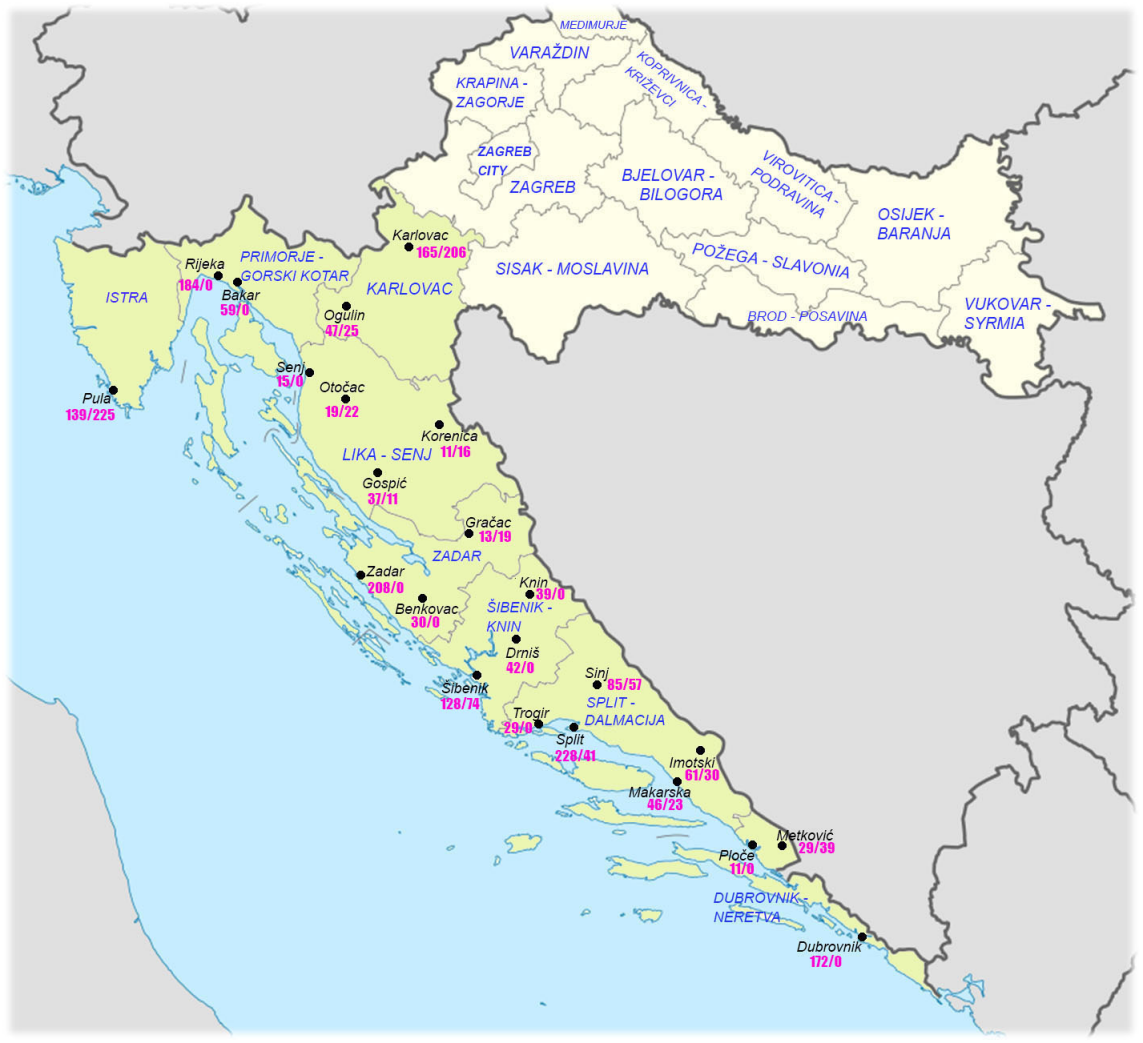
Political division of Croatia (counties /*županije*) with localities in which the measurements took place. Targeted counties are highlighted in green. Numbers designate the number of males/females measured in the schools of each town.

Adriatic Croatia consists of seven counties *(županijas)*: Istra, Primorje-Gorski Kotar, Lika-Senj, Zadar, Šibenik-Knin, Split-Dalmacija a Dubrovnik-Neretva. The total population in these seven counties is 1.41 million (33% of the total Croatian population) (Croatian Bureau of Statistics, 2013). Four counties of ‘Dalmatia proper’ include 0.86 milion people (20% of the total Croatian population). Because the counties of Primorje-Gorski Kotar and Lika-Senj are largely separated by the mainland county of Karlovac, we also decided to include the latter to have a more complete picture (Fig. 1). The eight counties together comprise 1.54 million people (36% of the total Croatian population).

The measured individuals consisted of high schoolers (3rd and 4th graders) aged 17–20 years, but whenever possible, we preferred young adults aged 18–20 years, in which we can assume a completed physical growth. Similar to our previous study in BiH, we always tried to measure the broadest spectrum of schools, from vocational to elite high schools *(gimnazija)*. Our general goal was to incorporate samples of at least 200 individuals from each region. This number is generally regarded as sufficiently representative, based on the experience of researchers participating in Czech anthropological surveys of school children (J Vignerová, pers. comm., 2013). For organizational or time reasons, it was not always possible to measure a sufficiently large sample of both sexes and hence we again concentrated mainly on males. The same limitations infuenced the measurement of body proportions.

### Data collection

The research was performed between April 2015–May 2017. For reasons of representativity, we tried to keep the ratio between elite high schools *(gimnazije)* and other school types in each county constant. The category ‘other schools’ can further be subdivided into the category ‘mixed high schools’ *(srednje škole)* that usually consist of multiple educational programs, including *gimnazije*. Very few of the schools contacted refused to participate, which allowed us to meet most of our goals. However, in contrast with BiH, where high school education is compulsory up to the age of 19 years, high school education in Croatia ends at the age of 18 years (or, in the case of some vocational schools, at the age of 17 years). This markedly reduced the number of students who were available at the appropriate age, when physical growth is already finished. Furthermore, *gimnazije* tend to be undersampled in most counties because of organizational issues and the worse availability of students preparing for the leaving exam.

The measurements were conducted using two devices—a mobile stadiometer SECA 213 and a specially constructed apparatus designed for the measurement of height, sitting height and arm span. The mutual compatibility and accuracy of these devices was tested on a sample of 128 Czech high schoolers. The means measured by these two devices differed by only 0.14 cm (179.18 cm vs. 179.04 cm), and their intra-class correlation was nearly perfect (*r* = 0.998).

Before the measurements, the students completed a short questionnaire, which included an informed consent agreement and questions about date of birth, place of residence and the level of education of the students’ parents. The measurements were performed according to standard anthropometric manuals, i.e., with the examined subjects standing/sitting erect, looking straight ahead, and with maximally extended arms. The results obtained during the survey were subsequently analyzed using the software Statistica 12 and SPSS 24. The values of height for each sample were described using means, standard deviations, medians, a range of extreme values, skewness, and kurtosis. In addition, three different tests of normal distribution were used for mutual comparison (Kolmogorov–Smirnov, Lilliefors and Shapiro–Wilk).

## Results

Altogether, the research included measurements of body height in 2,619 individuals (1,827 males and 792 females) at 66 high schools in 23 towns. This represents 38.7% of all schools that were available in the eight counties (Table S1). Results of the individual schools are presented in Table S2 . The proportion of students, who did not want to be measured was very small (<1%), so the samples can be regarded as highly representative of the school institutions in which they were conducted. Out of the measured sample, 1,803 males and 782 females were incorporated into this study, because they listed a place of residence in the targeted eight counties (Supplemental Dataset, Sheets 1–2). Their age ranged between 17.1–20.8 years with a mean of 18.5 years in males and 18.6 years in females. The males were born between 1995–2000, the females between 1996–2000. The excluded males and females (*n* = 34) came mostly from other Croatian counties, particularly from Sislak-Moslavina (*n* = 9) and Zagreb (*n* = 7), but some resided in BiH (*n* = 11) (see Supplemental Dataset, Sheets 1–4).

### Male height

**Figure 2:**
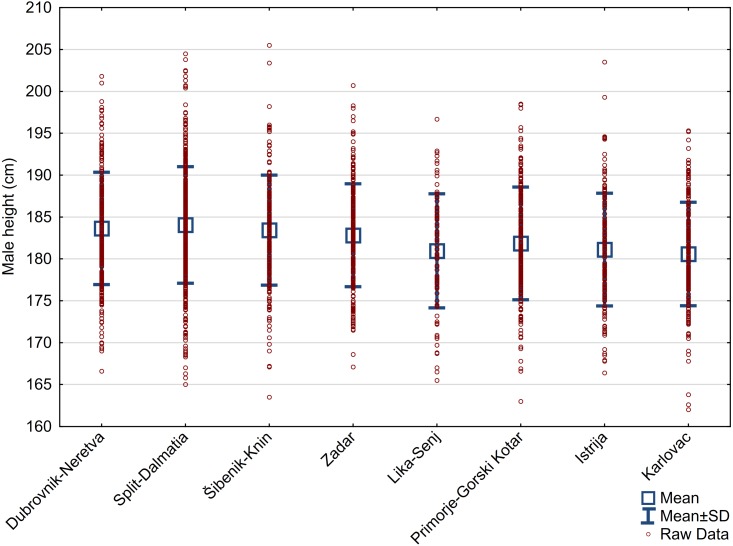
Range of individual values in eight counties.

The mean height of 1,803 males residing in the eight examined counties was 182.7 ± 6.7 cm. This mean did not change much when it was weighted for population size in these eight counties (182.6 cm) (Table S3). In the seven counties of Adriatic Croatia, a weighted male mean is only negligibly higher (182.8 cm), but it reaches 183.7 cm in the four counties of Dalmatia proper. Regional differences were large (3.5 cm), from 180.6 cm in the northernmost county of Karlovac to 184.1 cm in the county of Split-Dalmacija (Table 1, Fig. 2). It is noteworthy that boys aged 17 years (183.6 ± 6.7 cm, *n* = 245) are taller than boys aged 18–20 years (182.6 ± 6.7 cm, *n* = 1558), but this must be ascribed to the predominance of 17-year olds from the tallest Dalmatian regions. There was no evidence that any of these samples came from a population with a non-normal distribution of height (Table 2).

**Table 1:** Mean male height in individual counties *(županije)* according to the self-reported place of residence.

County *(županija)*	*n*	Age (years)		Male height (cm)				
		Mean (SD)	Minimum Maximum	Mean (SD)	Minimum Maximum	Median	Above 190 cm (%)	Above 200 cm (%)
Split-Dalmacija	*438*	18.3 ± 0.4	17.5–19.8	184.1 ± 6.9	165.0–204.5	183.9	79 (18.0%)	10 (2.3%)
Dubrovnik-Neretva	*241*	18.4 ± 0.4	17.5–20.1	183.6 ± 6.7	166.6–201.8	183.3	40 (16.6%)	2 (0.8%)
Šibenik-Knin	*209*	18.5 ± 0.4	17.2–19.7	183.4 ± 6.6	163.5–205.5	183.8	26 (12.4%)	2 (1.0%)
Zadar	*249*	18.4 ± 0.4	17.3–20.2	182.8 ± 6.2	167.1–200.7	182.8	31 (12.4%)	1 (0.4%)
Primorje-Gorski Kotar	*238*	18.7 ± 0.4	17.6–20.4	181.9 ± 6.7	163.0–198.5	181.8	29 (12.2%)	0
Istra	*143*	18.6 ± 0.4	17.2–20.7	181.1 ± 6.7	166.4–203.5	180.7	15 (10.5%)	1 (0.7%)
Lika-Senj	*86*	18.6 ± 0.4	17.5–20.2	181.0 ± 6.8	165.5–196.7	181.2	8 (9.3%)	0
Karlovac	*199*	18.7 ± 0.5	17.2–20.5	180.6 ± 6.2	162.0–195.3	180.8	13 (6.5%)	0
**TOTAL**	***1,803***	**18.5 ± 0.6**	**17.2–20.7**	**182.7 ± 6.7**	**162.0–205.5**	**182.7**	**241 (13.4%)**	**16 (0.9%)**

**Table 2:** Skewness, kurtosis and tests of normal distribution of male height in individual counties *(županije)*.

**County*(županija)***	***n***	**Mean male height (SD)**	**Skewness**	**Kurtosis**	**K-S test**	**Lillefors test**	**Sh-W test**
Split-Dalmacija	*438*	184.1 ± 7.0	0.08	0.30	*p* > 0.20	*p* > 0.20	*p* = 0.13
Dubrovnik-Neretva	*241*	183.6 ± 6.7	0.15	−0.24	*p* > 0.20	*p* < 0.15	*p* = 0.65
Šibenik-Knin	*209*	183.4 ± 6.6	−0.01	0.68	*p* > 0.20	*p* > 0.20	*p* = 0.27
Zadar	*249*	182.8 ± 6.2	0.11	−0.27	*p* > 0.20	*p* > 0.20	*p* = 0.43
Primorje-Gorski Kotar	*238*	181.9 ± 6.7	−0.02	−0.24	*p* > 0.20	*p* > 0.20	*p* = 0.73
Istra	*143*	181.1 ± 6.7	0.34	0.20	*p* > 0.20	*p* > 0.20	*p* = 0.37
Lika-Senj	*86*	181.0 ± 6.8	−0.18	−0.45	*p* > 0.20	*p* > 0.20	*p* = 0.64
Karlovac	*199*	180.6 ± 6.2	−0.10	0.00	*p* > 0.20	*p* > 0.20	*p* = 0.38
**TOTAL**	***1,803***	**182.7 ± 6.8**	**0.08**	**0.08**	***p*** > 0.20	***p*** > 0.20	***p*** = 0.30

**Notes.**

Abbreviations K-S test Kolmogorov–Smirnov test Sh-W test Shapiro–Wilk test

**Figure 3:**
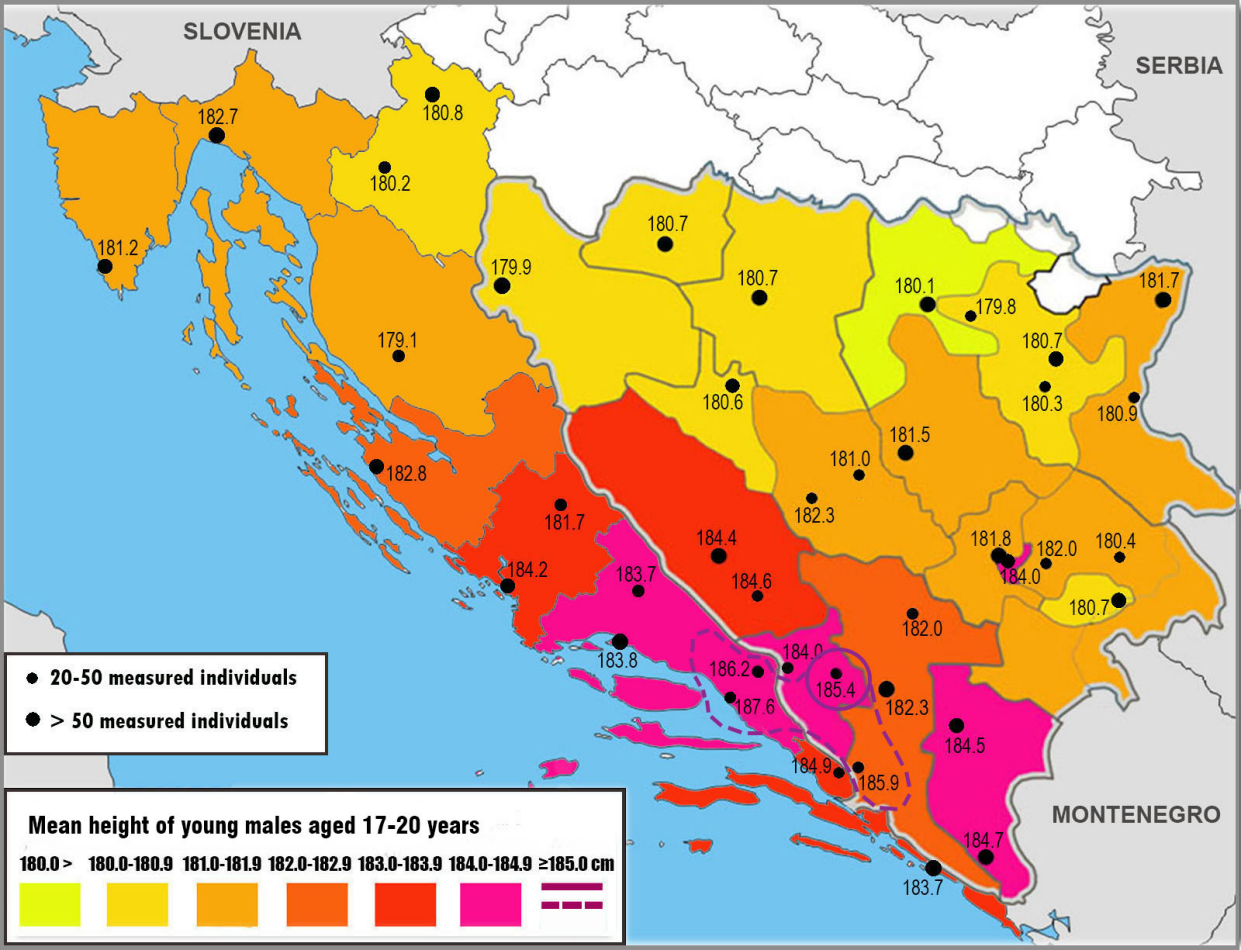
Regional means of male height on the Adriatic coast of Croatia and in Bosnia and Herzegovina, including mean male height in individual towns. All means are based on the self-reported place of residence (Tables 1 and 3).

**Table 3:** Mean male and female height in 14 individual towns according to the self-reported place of residence. Only towns with at least 20 measured individuals are included.

**County*(županija)***	**Town**	**Males**		**Females**		**Sex difference (cm)**
		*n*	Mean height (cm) (SD)	*n*	**Mean height (cm)** (SD)	
Split-Dalmacija	Makarska	*27*	187.6 ± 6.2			
Split-Dalmacija	Imotski	*21*	186.2 ± 8.3			
Dubrovnik-Neretva	Metković	*39*	184.9 ± 6.5	*27*	168.7 ± 7.3	16.2
Šibenik-Knin	Šibenik	*51*	184.2 ± 6.8	*32*	167.3 ± 4.6	16.9
Split-Dalmacija	Split	*154*	183.8 ± 7.2			
Dubrovnik-Neretva	Dubrovnik	*135*	183.7 ± 6.6			
Split-Dalmacija	Sinj	*34*	183.7 ± 7.8			
Zadar	Zadar	*90*	182.8 ± 5.5			
Primorje-Gorski Kotar	Rijeka	*118*	182.7 ± 6.5			
Šibenik-Knin	Knin	*45*	181.7 ± 5.9			
Istra	Pula	*97*	181.2 ± 6.9	*168*	166.8 ± 6.7	14.4
Karlovac	Karlovac	*97*	180.8 ± 6.0	*114*	166.6 ± 6.3	14.2
Karlovac	Ogulin	*33*	180.2 ± 6.0	*22*	167.7 ± 5.1	12.5
Lika-Senj	Gospić	*22*	179.1 ± 7.4			

### Geographical variation in male height

At first glance, the north-to-south trend of increasing height on both sides of the Croatian and Bosnian-Herzegovinian border is strikingly similar (Fig. 3). The the tallest counties of Dubrovnik-Neretva (183.6 cm), Split-Dalmacija (184.1 cm), and Šibenik-Knin (183.4 cm) make up a special group, and their means largely mirror those documented by our previous study in the adjacent regions of Herzegovina –Canton 10/Livno (183.7 cm), Canton Western Herzegovina (184.0 cm), Canton Herzegovina-Neretva (182.8 cm) and the region of Trebinje (184.5 cm). The unusually tall statures that we previously measured in the Herzegovinian towns of Čapljina (185.9 cm) and Široki Brijeg (185.4 cm) also have an analogy on the Croatian side of the border in the towns of Metković (184.9 cm), Imotski (186.2 cm) and Makarska (187.6 cm) (Table 3). Although all the samples are relatively small (21–38 males), such a high frequency of extremely tall means within a small geographical area cannot be a mere coincidence. This suggests that the peak of male height should be sought in the area demarcated by Makarska–Imotski–Široki Brijeg–Čapljina–Metković. However, school means of Croatian boys in Makarska (184.5 cm, *n* = 46), Imotski (184.3 cm, *n* = 55) and Metković (184.5 cm, *n* = 58), which better reflect the surroundings of these towns, indicate that this remarkable height is restricted to urban populations in this area. This applies even for the school in Čapljina in BiH (184.8 cm, *n* = 22). The tallest school mean in Dalmatia was actually documented in Sinj (184.6 cm, *n* = 85). Only two schools in Široki Brijeg exceed 185 cm (185.2 cm, *n* = 75).

In the direction to the north of this area, height begins to decrease quite rapidly, although this change is not always evident at the level of individual towns. For example, the mean of the Šibenik-Knin county (183.4 cm) is 0.7 cm lower than in the Split-Dalmacija county, but boys resident in Šibenik are still very tall (184.2 cm), even slightly taller than boys resident in Split (183.8 cm). A similarly high mean was found in the school in Drniš (184.4 cm, *n* = 42), which is situated approximately 35 km far inland in the Šibenik-Knin county. However, two schools in Knin, which is approximately 25 km further inland, were much shorter (182.8 cm, *n* = 39) and a sample of boys resident in Knin reached only 181.7 cm. Shorter statures in inland Dalmatia were previously reported even by Prebeg (1988) and ascribed to economic factors.

Height further decreases by 0.6 cm in the county of Zadar (182.8 cm). It again appears to be more pronounced inland, as indicated by the low mean measured in the school of Gračac (178.8 cm, *n* = 13). Still, it should be mentioned that the area of Gračac is very sparsely populated and it is very difficult to collect samples which would be adequately representative. In addition, the local population consists of many Croatian immigrants from BiH, who may not have the same genetic background.

The drop in height is relatively the most dramatic in the county of Lika-Senj (by 1.8 cm to 181.0 cm), which separates Dalmatia from the rest of Croatia. However, considering that there are only five high schools in this sparsely populated and mountainous region, the final result may have been influenced by the absence of Gimnazija Gospić that refused to participate in the survey. In fact, the mean of the town of Gospić, based overwhelmingly on boys from the local vocational school *(Strukovna škola)* was only 179.1 cm. Understandably, boys from a vocational school represent the bottom end of the social spectrum, while boys from *gimnazija* represent the upper end, and hence the mean of the whole county may be underestimated. If we exclude boys from Strukovna škola Gospić, the mean of boys resident in the Lika-Senj county would be 181.9 cm (*n* = 49), which is in line with the mean of the Primorje-Gorski Kotar county (181.9 cm) bordering the northwest.

It is noteworthy that 118 boys residing in Rijeka—the capital of the Primorje-Gorski Kotar county—were quite tall (182.7 cm), matching their peers in northern Dalmatia. Other towns were not represented by a sufficient number of boys, but at least those from neighbouring Viškovo (181.4 cm, *n* = 16) conform to the usual height standard in this region.

In the county of Istra (the Istria peninsula), height decreases by 0.8 cm to 181.1 cm, although this result has certain limitations, because it was based on eight schools from a single town (Pula). However, the total picture indicates that height means remain quite high (>181 cm) in all the coastal areas surrounded by the mountain ridges of the Dinaric Alps. Across the Velika Kapela ridge, the height mean falls quite markedly, from 181.9 in the Primorje-Gorski Kotar county to 180.6 cm in the mainland county of Karlovac. This is not very different from the mean of boys from Zagreb (180.1 cm) mentioned in the introduction to this paper.

**Table 4:** Linear mixed effect model of five geographical and socioeconomic variables in which attended school and the place of residence were selected as random effects (a sample of 1,708 males for which all data were available). Individual counties are ranked according to the latitude of their capitals.

Parameter	Estimate (height values in cm)	95% Confidence interval	*p*-value
**Intercept (constant)**	**180.6**	**179.7, 181.6**	**<0.001**
School Type: Gimnazija	0.56	−0.24, 1.37	0.17
School Type: Mixed	0.76	−0.19, 1.71	0.12
School Type: Other	0^a^		
Age: 17.0–17.9 years	0.30	−0.68, 1.27	0.55
Age: 18.0 + years	0^a^		
County: DUBROVNIK-NERETVA	2.81	1.55, 4.07	**<0.001**
County: SPLIT-DALMACIJA	3.19	1.99, 4.38	**<0.001**
County: ŠIBENIK-KNIN	2.89	1.55, 4.24	**<0.001**
County: ZADAR	2.32	1.07, 3.58	**<0.001**
County: ISTRA	0.32	−1.12, 1.77	0.66
County: LIKA-SENJ	0.45	−1.29, 2.19	0.61
County: PRIMORJE-GORSKI-KOTAR	1.29	0.04, 2.55	**0.044**
County: KARLOVAC	0^a^		
Urban residence: NO	−0.66	−1.34, 0.02	0.057
Urban residence: YES	0^a^		
University education of parents: BOTH	0.20	−0.78, 1.19	0.69
University education of parents: ONE	−0.12	−0.87, 0.64	0.76
University education of parents: NEITHER	0^a^		

**Notes.**

Significant values are highlighted in red. The sum of values from individual categories can predict mean male height for the particular combination of factors, relative to the intercept (180.7 cm). For example, males from the county of Split-Dalmacija (+3.19 cm), aged 18 + years (0 cm), from elite high schools *(gimnazija)* (+0.56 cm), urban localities (0 cm), and with university-educated parents (+0.20 cm) would be 184.6 cm tall.

a This parameter represents a standard within the particular category and is set to zero.

**Table 5:** Differences in mean male height (cm) across eight counties (above the diagonal) and confidence intervals (below the diagonal), according to a mixed effect model using the county of residence, university education of parents, urban/rural residence, school type and age categories as fixed effects, and attended school and the place of residence as random effects. Significant differences between regions are highlighted in orange.

	**Dubrovnik-Neretva**	**Split-Dalmacija**	**Šibenik-Knin**	**Zadar**	**Istra**	**Lika-Senj**	**Primorje-Gorski Kotar**	**Karlovac**
**Dubrovnik-Neretva**		**0.38**	**0.08**	**−0.49**	**−2.49**	**−2.36**	**−1.52**	**−2.81**
**Split-Dalmacija**	**(−0.72, 1.48)**		**−0.29**	**−0.86**	**−2.86**	**−2.74**	**−1.89**	**−3.19**
**Šibenik-Knin**	**(−1.19, 1.36)**	**(−1.49, 0.90)**		**−0.57**	**−2.57**	**−2.44**	**−1.60**	**−2.89**
**Zadar**	**(−1.69, 0.71)**	**(−1.97, 0.24)**	**(−1.82, 0.68)**		**−2.00**	**−1.87**	**−1.03**	**−2.32**
**Istra**	**(−3.87, −1.10)**	**(−4.21, −1.52)**	**(−4.05, −1.09)**	**(−3.40, −0.60)**		**0.13**	**0.97**	**−0.32**
**Lika-Senj**	**(−4.05, 0.67)**	**(−4.37, −1.11)**	**(−4.14, −0.75)**	**(−3.52, −0.22)**	**(−1.72, 1.97)**		**0.84**	**−0.45**
**Primorje-Gorski Kotar**	**(−2.72, −0.31)**	**(−3.04, −0.74)**	**(−2.89, −0.31)**	**(−2.22, 0.17)**	**(−0.41, 2.35)**	**(−0.85, 2.54)**		**−1.29**
**Karlovac**	**(−4.07, −1.55)**	**(−4.38, −1.99)**	**(−4.24, −1.55)**	**(−3.56, −1.07)**	**(−1.77, 1.12)**	**(−2.19, 1.29)**	**(−2.55, −0.04)**	

**Notes.**

Level of significance:
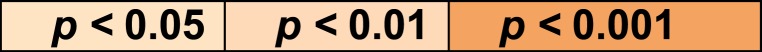

### Linear mixed effect model of geographical and socioeconomic variables

Because some categories in our dataset (e.g., school type and the place of residence) cannot be regarded as fully independent, and high schoolers from the same school or town may share certain characteristics that distinguish them from other schools and towns, we tested a linear mixed effect model in which the county of residence, university education of parents (both/one/neither), school type (*gimnazija*, mixed school, other schools), urban/rural residence (above/below 10,000 inhabitants), and age categories (17.0–17.9 years and 18.0 + years) were taken as fixed effects, and attended school and the place of residence were random effects. Tables 4–5 show that, at least in boys, the documented differences between northern and southern counties are highly significant and persist even in the mixed effect model, reaching 3.2 cm between counties of Karlovac and Split-Dalmacija. Differences in other categories are not significant.

### Female height

The mean height of 782 girls from 28 schools was 167.4 ± 6.2 cm. Regional differences were relatively large (2.5 cm), but lower than in males: from 166.6 cm in the county of Karlovac to 169.1 cm in the county of Dubrovnik-Neretva (Table 6). Nevertheless, only samples from the counties of Istra, Karlovac and Split-Dalmacija incorporated more than 100 girls (*n* = 148–225), whereas four other counties were represented by fewer than 50 individuals. Only five girls came from the Primorje-Gorski Kotar county. Therefore, the informative value of female samples is clearly more limited. Still, all of them have a normal distribution of values.

**Table 6:** Mean female height in individual counties *(županije)* accoding to the self-reported place of residence.

**County*(županija)***	***n***	**Age (years)**		**Female height (cm)**		**Mean male height (cm)(SD)**	**Difference(men-women)**	**Male–female ratio**
		**Mean (SD)**	**MinimumMaximum**	**Mean (SD)**	**MinimumMaximum**			
Split-Dalmacija	*148*	18.5 ± 0.5	17.8–20.4	168.9 ± 5.9	151.5–186.0	184.1 ± 6.9	15.2	1.090
Dubrovnik-Neretva	*39*	18.4 ± 0.2	18.0–19.0	169.1 ± 7.1	156.2–188.0	183.6 ± 6.7	14.5	1.086
Šibenik-Knin	*74*	18.9 ± 0.6	17.9–19.9	167.9 ± 5.1	156.0–179.2	183.4 ± 6.6	15.5	1.092
Zadar	*20*	18.4 ± 0.5	17.3–19.1	167.5 ± 6.9	151.4–183.0	182.8 ± 6.2	15.3	1.091
Primorje-Gorski Kotar	*5*	18.4 ± 0.3	18.1-18.8	168.8 ± 5.5	161.7-178.3	181.9 ± 6.7	13.1	1.078
Istra	*225*	18.5 ± 0.5	17.1–20.8	166.7 ± 6.7	148.2–182.6	181.1 ± 6.7	14.4	1.086
Lika-Senj	*48*	18.5 ± 0.4	18.0–20.1	168.0 ± 5.4	152.3-180.2	181.0 ± 6.8	13.0	1.077
Karlovac	*223*	18.5 ± 0.4	17.5–20.0	166.6 ± 6.0	151.2-181.3	180.6 ± 6.2	14.0	1.084
**TOTAL**	***782***	**18.6 ± 0.5**	**17.1–20.8**	**167.4 ± 6.2**	**151.2–188.0**	**182.7 ± 6.7**	**15.3**	**1.091**

After the correction for population size, excluding the weakly represented county of Primorje-Gorski Kotar, the mean female height would be 168.5 cm in Dalmatia, 168.2 cm in six counties of Adriatic Croatia and 168.0 cm in all seven counties. In general, it seems that girls in counties with the tallest boys (around 184 cm) reach a height of approximately 169 cm.

Using a similar mixed effect model as for boys, but without the county of Primorje-Gorski Kotar and without parental education (which was missing in 18.9% girls), the range of values decreases from 2.5 cm to 1.4 cm (between counties Dubrovnik-Neretva and Šibenik-Knin), and does not reach significance (*p* = 0.55; 95% CI [ −3.2–6.0]).

### Relationship between male and female height

The sex difference in mean height in our study is unusually high, reaching 15.5 cm in the county of Šibenik-Knin, and 15.3 cm between the total samples of boys and girls (Table 6). Even if we compare samples that have the largest sizes (Istra, Karlovac), the sex difference is still 14.4 and 14.0 cm, respectively. At the same time, the usual difference in Europe is 13 cm (Steckel, 1996). The gap is only 13.1 cm even in the world’s tallest nation—the Dutch.

Table 7 summarizes male and female heights in eight countries/territories that extend to the Dinaric Alps. The values strongly correlate with each other ( *r* = 0.99, *p* < 0.001), but the sex-related discrepancy is similarly high, reaching 14.5 cm in Serbia, and it tends to increase with the increasing population mean. According to our unpublished data from 142 countries/territories (in review), this disproportionate relationship between male and female height exists even at the global level. Male and female statures in this global sample strongly correlate (*r* = 0.97; *p* < 0.001), but both the male–female difference and male–female ratio in tall nations increases. Female height can be predicted via an equation “Female height = 11.125 + (0.8637 * Male height)“. The expected sex difference would be 14 cm at the male height of 184 cm (a ratio of 1.082), 13.7 cm at 182 cm (1.081), and 13.4 cm at 180 cm (1.080). Therefore, the extraordinary tallness documented in some Dinaric regions can also be responsible for the greater sexual dimorphism, but the documented differences still appear to be above-average in the global context.

**Table 7:** Differences in the mean height of males and females on the territory of the Dinaric Alps.

**Country/region**	**Date**	**Age**	**Mean height (cm)**		**Sex differ. (cm)**	**Male–female ratio**	**Source**
			**Males**	**Females**			
Serbia	2013	20–25	181.2	166.7	14.5	1.087	I Ivanović, pers. comm. 2016
BiH (Federation)	2012	20–25	182.2	167.9	14.3	1.085	A Pilav, pers. comm. 2015
Croatia	2006-08	18	180.5	166.3	14.2	1.085	Jureša, Musil & Kujundžić Tiljak (2012)
Montenegro	2013	17–20	183.4^a^	169.4^a^	14.0	1.083	Popović (2017)
Kosovo	2016	17–18	179.5	165.7	13.8	1.083	Popović, Arifi & Bjelica (2017)
Slovenia	2012	18–21	179.8	166.5	13.3	1.080	G Starc, pers. comm. 2013
Macedonia	2012	18	177.4	164.5	12.9	1.078	Gontarev et al. (2014)
Albania	2008-09	20–29	174.0	161.8	12.1	1.075	IS & IPH Albania & ICF Macro (2010)

**Notes.**

a These values would decrease to 182.9 cm and 168.8 cm, respectively, if we took population size in individual regions into account.

### Body proportions

Information on body proportions is available for only 209 boys. The majority (*n* = 121) came from the northern part of the Adriatic coast (counties Istra, Primorje-Gorski Kotar and Karlovac). The remaining ones (*n* = 88) resided in the southern part (counties Dubrovnik-Neretva and Split-Dalmacija) and were measured at Pomorska škola in Dubrovnik. Despite this limitation, the results are interesting, because when combined with our previous data from BiH, they indicate geographical trends in body proportions (Table 8).

**Table 8:** Mean height and body proportions in males on the Adriatic coast of Croatia and in Bosnia and Herzegovina (according to the self-reported place of residence).

**Age (years)**	***n***	**Mean height (cm)(SD)**	**Sitting height (cm) (SD)**	**Sitting height(% height)(SD)**	**Arm span (cm)(SD)**	**Arm span(% height)(SD)**
Bosnia	*1399*	180.7 ± 6.7	94.8 ± 3.3	52.48 ± 1.30	181.9 ± 7.7	100.6 ± 2.2
Adriatic coast (North)^a^	*121*	180.5 ± 5.9	94.4 ± 3.0	52.32 ± 1.32	183.5 ± 8.1	101.6 ± 2.7
Adriatic coast (South)^b^	*88*	184.3 ± 6.6	95.9 ± 3.4	52.04 ± 1.30	184.8 ± 7.6	100.3 ± 2.4
Herzegovina	*451*	184.1 ± 7.2	95.9 ± 3.4	52.11 ± 1.63	184.6 ± 8.3	100.3 ± 2.3

**Notes.**

a Counties Istra, Primorje-Gorski Kotar and Karlovac.

b Counties Dubrovnik-Neretva and Split-Dalmacija.

The boys from the southern region have height and body proportions that are virtually indistinguishable from boys in neighbouring Herzegovina—tall stature around 184 cm, low relative sitting height (52.0–52.1% body height) and hence relatively longer legs, but relatively short arm span (100.3% body height). Older data from Montenegro (see Coon, 1939, p. 592) report a very similar values of relative sitting height (52%) and arm span (101%). Anthropometric characteristics of boys from the northern region and from Bosnia are mutually similar as well –much shorter height (180.5–180.7 cm), higher relative sitting height (52.3–52.5%) and higher relative arm span (100.6–101.6%).

It is well known that relative sitting height decreases with increasing stature (Bogin et al., 2002). In fact, the difference in height between shorter northern and taller southern regions is mostly due to leg length. In contrast, relative arm span decreases with increasing height. Therefore, we can expect that at the same mean height, there would be no differences in body proportions across different regions of the Dinaric Alps. Nevertheless, there may exist noticeable differences among populations from more distant regions of Europe (Coon, 1939).

Information on body proportions was also collected in 171 girls coming from the counties of Istra, Primorje-Gorski Kotar and Karlovac. Their height was 166.2 ± 6.6 cm, relative sitting height 53.4 ± 1.5%, and relative arm span 99.9 ± 2.2%. The fact that women have relatively shorter limb length than men is well documented (Norton & Olds, 1996).

### Parental education

Similar to our previous study in BiH, we collected information on parental education. Altogether, 1,708 boys and 634 girls reported the education of their parents in questionnaires. Out of this number, 60.8% boys and 63.7% girls reported that none of their parents have university education, but 13.6% boys and 12.5% girls had both parents with university education. The percentages of boys and girls in each category of parental education are very similar (Table 9) which would indicate that these self-reported data are reliable. However, the self-reported percentage of university-educated parents is almost always higher than the true proportion of university-educated adults aged 35–39 years or 40–44 years (for 2011), as provided by the Croatian Bureau of Statistics (http://www.dzs.hr) (Table S4). Thus, students probably systematically overreported the university education of their parents. This assumption is further supported by the fact that in the category of 40-44 years, these self-reported data correlate fairly well with the true proportion of adults with all forms of higher education (*r* = 0.81, *p* = 0.015 in boys; *r* = 0.66, *p* = 0.08 in girls), but much less with the true proportion of adults with university education *sensu stricto* (*r* = 0.68, *p* = 0.061 in boys; *r* = 0.53, *p* = 0.18 in girls).

**Table 9:** Relationship between the height of measured males & females and the university education of their parents.

**Parental education (university)**	**Males**			**Females**			**Both sexes**	
	***n***	***% total***	**Mean height (cm)**	***n***	***% total***	**Mean height (cm)**	***n***	**% total**
Neither parent	1,038	*60.8*	182.6	404	*63.7*	167.0	1,442	*61.6*
Father	459	*26.9*	182.8	155	*24.4*	167.6	614	*26.2*
Father only	226	*13.2*	182.5	76	*12.0*	167.1	302	*12.9*
Mother	443	*25.9*	182.8	154	*24.3*	167.9	597	*25.5*
Mother only	211	*12.4*	182.5	75	*11.8*	167.7	286	*12.2*
One parent	438	*25.6*	182.5	151	*23.8*	167.4	589	*25.1*
Both parents	232	*13.6*	183.1	79	*12.5*	168.1	311	*13.3*

In contrast with the results in BiH where we found a difference of 1.9 cm between males in categories “both parents” and “neither parent”, in the present study we documented a much smaller disparity in this regard (0.5 cm: 183.1 ± 7.1 cm vs. 182.6  ± 6.7 cm). In the mixed effect model, this difference decreases to 0.2 cm and it is not significant (*p* = 0.69, 95% CI [−0.8–1.2]) (Table 4). The differences between categories “both parents” and “neither parent” were somewhat more pronounced in girls (1.1 cm: 168.1 ± 6.5 cm vs. 167.0 ± 6.2 cm), but in a mixed effect model including 634 girls (without missing data on parental education), they did not reach significance either (1.2 cm; *p* = 0.14, 95% CI: -0.4, 2.8).

### Types of school

As expected, high schoolers attending elite schools *(gymnazije)* tended to be taller, and males from the tallest county Split-Dalmacija reached 184.6 cm on average (Table 10). In the shortest county Karlovac, they were 181.1 cm tall which exactly corresponds to the height gap observed at the county level. The percentage of boys who had both parents with university education was the highest at *gimnazije* (26.1%), and the lowest at ‘other’ types of schools (10.6%) and at ‘mixed schools’ (6.0%). Nevertheless, differences in male height among three predefined types of schools were generally small and were not significant in the mixed effect model (Table 4). The differences seem to be higher in girls, at least in some counties.

**Table 10:** Relationship between the height of measured males & females and the type of attended schools.

	**Gimnazije**		**Mixed schools**		**Other schools**	
	***n***	**Mean height (SD)**	***n***	**Mean height (SD)**	***n***	**Mean height (SD)**
**MALES (total sample)**	*405*	182.8 ± 7.0	*252*	183.4 ± 6.8	*1,146*	182.5 ± 6.6
Karlovac county	*42*	181.1 ± 6.2	*3*	184.6	*154*	180.4 ± 6.2
Dalmatia	*204*	184.2 ± 6.8	*197*	183.7 ± 6.8	*724*	183.3 ± 6.6
Split county	*62*	184.6 ± 7.1	*59*	184.5 ± 6.6	*317*	183.9 ± 7.0
**FEMALES (total sample)**	*231*	168.1 ± 6.5	*57*	167.6 ± 6.1	*494*	167.1 ± 6.1
Karlovac county	*73*	168.3 ± 6.1	*1*	153.6	*149*	165.8 ± 5.8
Dalmatia	*62*	169.7 ± 6.8	*18*	168.2 ± 6.9	*199*	168.3 ± 5.5

### Urban vs. rural origin

Differences across urban and rural populations are small as well. Urban boys from towns with 25,000+ inhabitants were 182.8 ±6.7 cm tall (*n* = 761), boys from towns and villages with fewer than 25,000 inhabitants reached 182.6 ± 6.8 cm (*n* = 1,042), boys from towns with >10,000 inhabitants 182.9 ± 6.8 cm (*n* = 1,061), and boys from towns and villages with fewer than 10,000 inhabitants 182.4 ± 6.7 cm (*n* = 742). When the cut-off point between urban boys and rural boys is set at 10,000 inhabitants, there is no significant difference between these two groups according to the mixed effect model (0.66 cm; *p* = 0.057, 95% CI [−1.3–0.00]). Urban girls (166.9 ± 6.5 cm, *n* = 464) were shorter than rural girls (168.1 ± 5.7 cm, *n* = 318), but in a mixed effect model incorporating 634 girls from all counties, the difference was not significant either (0.94 cm; *p* = 0.12, 95% CI [−0.2–2.1]).

### Monthly net salary and unemployment

In addition, we tested the relationship of male height in eight counties with the statistics of monthly net salary (in kuna/HRK) between 1995–2014, and unemployment between 1998–2014, provided by the Croatian Bureau of Statistics (https://www.dzs.hr). No significant trends can be observed (Table S5, Figs. S1–S4), and the inclusion of these two statistics into a regression model does not lead to any significant results either, explaining only ∼5% variance (Table S6).

However, it is noteworthy that eight out of top 11 Croatian counties with the highest net salaries belong to the Adriatic region that we examined in the present study. This must undoubtedly be attributed to the effect of tourism (see http://croatia.eu/article.php?lang=2&id=34). Therefore, the impact of economic factors could potentially manifest if we compared counties of the Adriatic region with the rest of Croatia. Similar to Prebeg (1988), we can also suppose that the shorter statures documented in the inland of Dalmatia are due to the fact that the economic benefits of tourism are limited to the Adriatic coast.

### Nutrition

**Figure 4:**
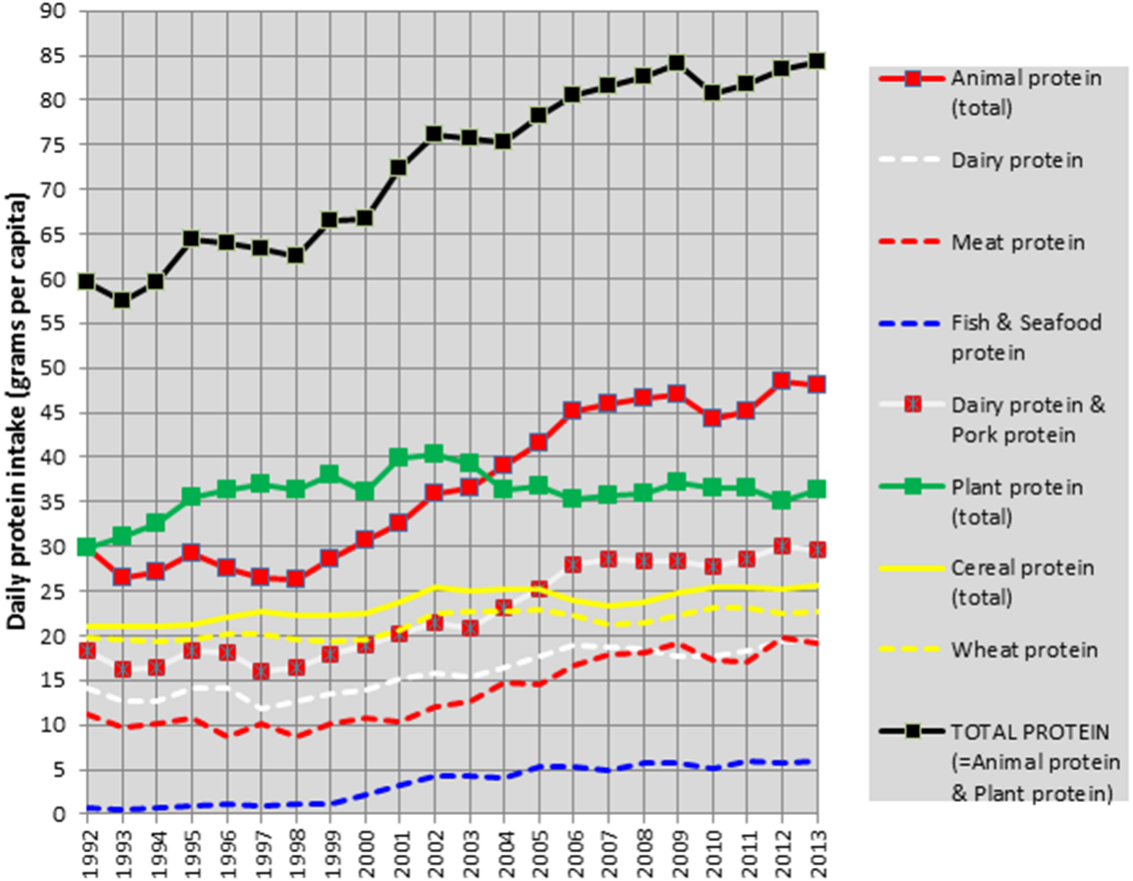
Mean daily protein intake (supply) from major protein sources in Croatia between 1992–2013. Source: FAOSTAT. Food supply. Crops Primary Equivalent. http://www.fao.org/faostat/en/#data/CC. Food supply. Livestock and Fist Primary Equivalent. http://www.fao.org/faostat/en/#data/CL.

The Croatian Bureau of Statistics was not able to provide any data on the regional food production or consumption, and hence our nutritional analysis depends only on crude national statistics of food supply from the FAOSTAT database. These data show that protein consumption in Croatia has dramatically increased since 1992 and most of this increase can be attributed to animal proteins (Fig. 4). The consumption of proteins from dairy and pork, which have the strongest positive connection with adult height in our ecological studies (Grasgruber et al., 2014; Grasgruber et al., 2016), is almost twice higher, when compared with 1993.

**Figure 5:**
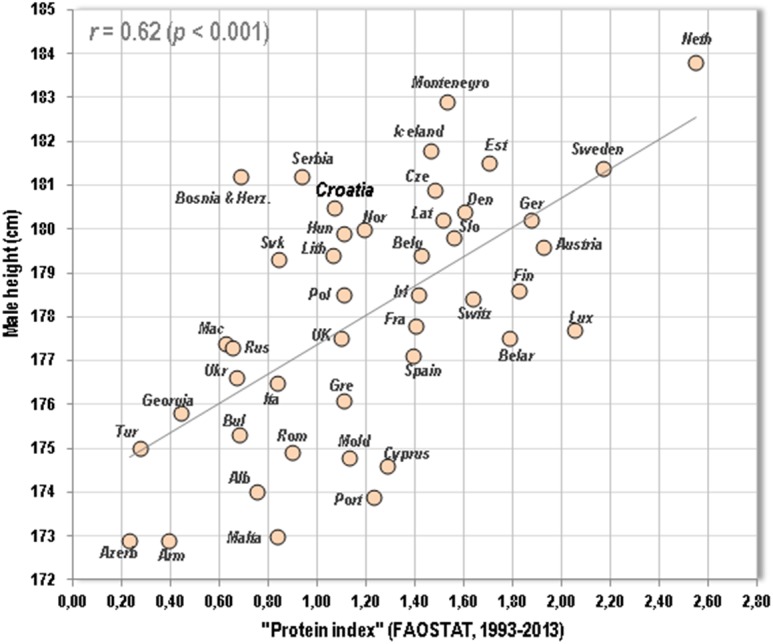
Relationship between mean male height and the “protein index” (the ratio between high-quality proteins from dairy and pork, and low-quality proteins from wheat) in 44 European countries (FAOSTAT, 1993–2013) Note: The graph contains updated values of male height.

However, this development apparently copies the trend of the GDP per capita, which decreased dramatically from 8907 USD in 1990 to 5803 USD in 1993 (−35%). The level of 1990 was surpassed as late as in 2002 (9026 USD) (The National Accounts Main Aggregates Database). Therefore, the current quality of nutrition in Croatia probably isn’t much higher than in the 1980s. Indeed, the ‘protein index’ (the ratio between high-quality animal proteins and low-quality plant proteins), which is the strongest dietary predictor of male height in Europe (Grasgruber et al., 2014), is still quite low in Croatia. In the present updated sample of 44 European countries, proteins from dairy & pork/wheat produce the highest correlation coefficient (*r* = 0.62, *p* < 0.001) (Fig. 5). The Croatian mean for the period 1993–2013 is 1.07, which is below the European mean (1.21). During the last decades, this index has increased only moderately, from 0.93 in 1992 to 1.30 in 2013, and has been basically stagnating since 2007 which can be connected with the onset of the economic crisis (2008). Therefore, when expressed by this index, the quality of nutrition in Croatia is still suboptimal, which is a situation that has analogies even in other countries of the former Yugoslavia (Bosnia and Herzegovina, Macedonia, Serbia). During the period 1993-2013, only Slovenia (1.56) and Montenegro (1.53) are above average in this regard

### Genetics

**Figure 6:**
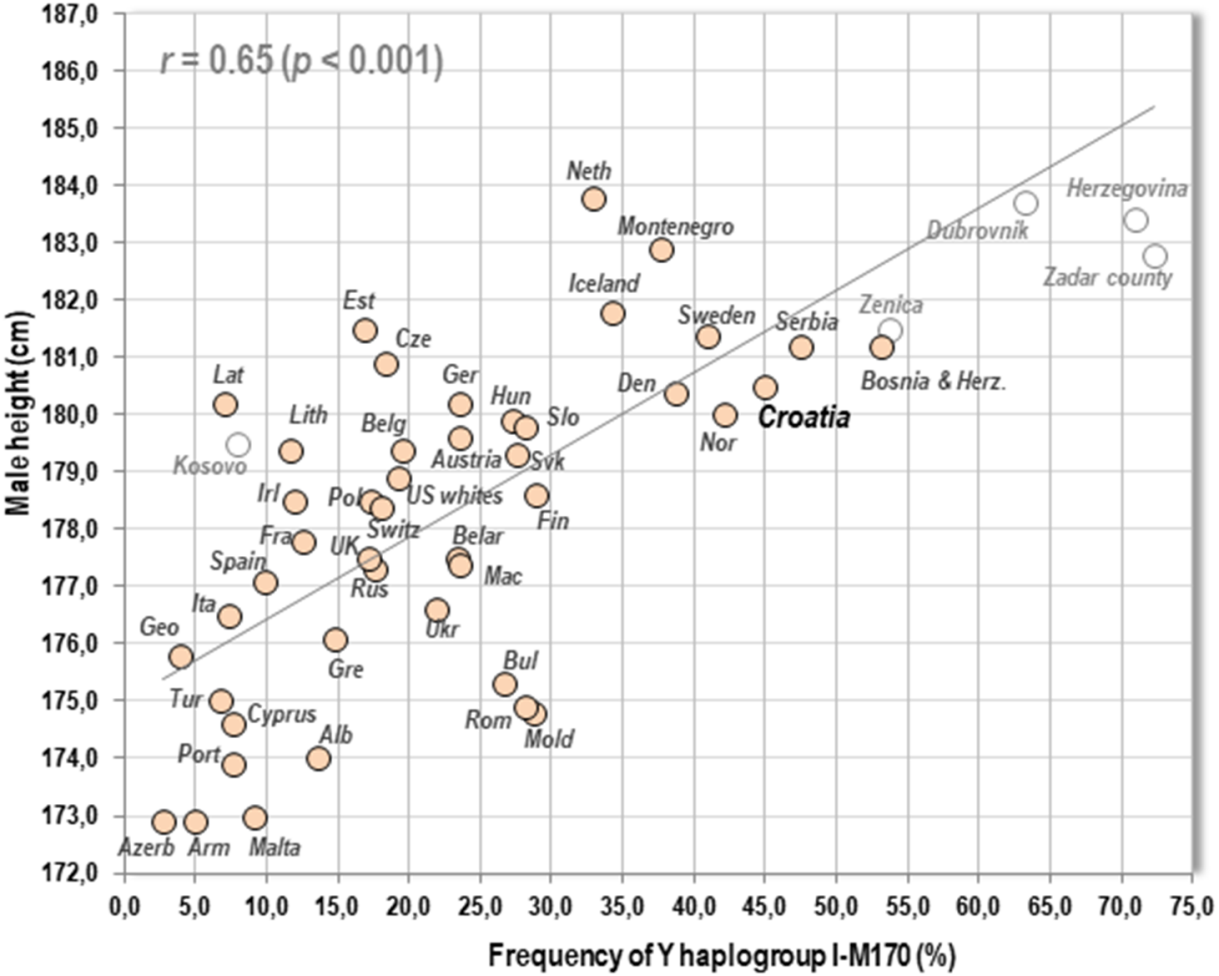
Relationship between mean male height and the frequency of Y haplogroup I-M170 in 44 countries of Europe & US whites. Five regional samples from the former Yugoslavia are displayed for additional comparison. Note: Regional I-M170 frequencies were taken from the following sources: for Zenica, Herzegovina, and Kosovo (Kosovar Albanians) from Pericic et al. (2005); for the Zadar county (‘Zadar hinterland‘) and Dubrovnik from Šarac et al. (2016).

The genetic aspects of the exceptional stature on the Adriatic coast are certainly the most interesting, and are supported by the geographical distribution of *Y* haplogroup I-M170 which shows a highly significant relationship with male stature in 44 European countries and the white US population (*r* = 0.65, *p* < 0.001) (Fig. 6). Although Y haplogroups are primarily used as markers of genealogical origin, they can also serve as signatures of separated patrilocal communities and ‘founder effects’. This explains why they correlate with the geographical distribution of language families (cf. Van Driem, 2011). In fact, even regional data on I-M170 frequencies from Herzegovina, Bosnia, the Zadar county and Dubrovnik (Pericic et al., 2005; Šarac et al., 2016) fit the trend between height and I-M170 at the country level, although height is still lower than expected. This can be attributed to suboptimal living conditions which still do not allow the maximum expression of the genetic potential. There is little doubt that we could expect values over 185 cm in Herzegovina and its close surroundings, provided that living standards reached those in Western Europe—and some town means already markedly exceed this figure.

Interestingly, I-M170 is not the only Y haplogroup in Europe showing a geographical association with height. A statistically non-significant positive correlation can also be found with Y haplogroup R1a-M420 (*r* = 0.24, *p* = 0.12) which currently dominates in Slavic speaking nations of Eastern Europe. In contrast, the combination of predominantly Neolithic Y haplogroups E-M96, G-M201 and J-M304 correlates negatively ( *r* =  − 0.65, *p* < 0.001). R1b-S116, the major *Y* haplogroup in Atlantic Europe, also tends to correlate negatively. The strongest predictor of height in Europe is the combination of I-M170 with R1b-U106, a typical *Y* haplogroup of Germanic speaking nations (*r* = 0.72, *p* < 0.001), but these data are still available from only 34 European countries.

Our previous study (Grasgruber et al., 2014) showed that genetic factors and nutrition are the two most important factors explaining geographical differences in body height across Europe. In a current work dealing with the determinants of height in 150 world countries (in review), male height in 42 European countries can best be predicted by the combination of I-M170 frequencies, the ‘protein index’ (dairy & pork/wheat proteins), the Human Development Index and the GDP per capita (adj. *R*^2^ = 0.688, *p* < 0.001). At the same time, the combination of I-M170 and the ‘protein index’ explains by far the largest part of this variation (adj. *R*^2^ = 0.615, *p* < 0.001).

## Discussion

To our knowledge, this study constitutes the most detailed report on the regional differences in stature on the Adriatic coast of Croatia that has been published since the end of the 19th century. As already mentioned above, these differences reached 5 cm (166–171 cm) at the end of the 19th century and this range has largely persisted until today, because we documented a 3 cm gap (181.1–184.1 cm) between the counties of Istra and Split-Dalmacija. At the same time, mean heights from the four counties of Dalmatia proper are among the highest that have ever been measured in a human population. In fact, Dalmatian boys aged 18 years (*n* = 839) are 183.7 cm tall (after controlling for population size in regions) which shows that they would be markedly taller than their peers in the Netherlands who reach 182.4 cm at the age of 18 years, and 183.6 cm at the age of 19 years (Schönbeck et al., 2012). The size of the Dutch samples is also quite small (only 180 males aged 18-19 years) and it is not entirely clear if the samples past high-school age (18+ years) are not artificially elevated due to the inclusion of university students (as mentioned in the ‘Methods’ section of that paper). According to Schönbeck et al. (2012), there are regional differences within the Netherlands as well, but the height of boys from the tallest northern regions is ca. 0.15 standard deviation above the mean, which is ca. 183.5 cm in 18-year olds. Besides that, Montenegrin boys may also be taller than their Dutch peers even at the country level.

The height of Dalmatian girls (168.5 cm) is based on limited data, and appears to be shorter than in the Netherlands where girls achieve 169.7 cm at the age of 18 years, and 170.1 cm at the age of 19 years. We found only one school with a mean over 170 cm - Gimnazija Imotski (170.5 cm, *n* = 24), and ten other Dalmatian schools were in the range of 167.0–169.4 cm.

In the global context, there is possibly only one population that could potentially compete with the Dutch and Dinaric people for the tallest in the world: South Sudanese Nilotes. Shortly after World War II, the height of men from various Nilotic tribes ranged between 178.7–184.9 cm (Twiesselmann & Nyèssen, 1951; Roberts & Bainbridge, 1963; Crognier, 1973), with the sample of Dinka Ruweng being the most representative (181.3 cm, *n* = 227). However, it seems that due to war and famine, their height has decreased during recent decades (Chali, 1995; Rebacz, 2006).

Another intriguing finding of our study is the striking similarity of height trends on both sides of the Croatian and Bosnian-Herzegovinian border. The existence of a north-to-south gradient can be expressed even statistically, as shown by the correlation between male height in the eight Croatian counties and the latitude of the county’s capital (*r* = 0.88, *p* = 0.004). Therefore, if any local factors influence stature, they must work similarly on both sides of the border.

Coincidentally, recent paleogenetic studies brought new evidence that this extraordinary tallness has deep roots going to the Upper Paleolithic. These studies show that Gravettian samples from Europe dated to the period 33,000–26,000 calibrated ^14^C years ago are genetically similar and make up a separate ‘Věstonice cluster’ (Fu et al., 2016; Sikora et al., 2017). No signal of genetic selection for height within these Gravettian populations can be detected, but a dramatic development of this sort occurs in individuals from a more recent ‘Villabruna cluster’ (Berg, Zhang & Coop, 2017). The oldest tested sample of this cluster (Villabruna in Northern Italy, ∼14 000 cal. ^14^C years ago) is associated with the Epigravettian culture (Fu et al., 2016) that was widespread in Italy and Southeastern Europe after the onset of the Last Glacial maximum, ca. 23,000–11,000 cal. ^14^C years ago (Naudinot et al., 2017).

The ‘Villabruna cluster’ is identical with the ‘Western European hunter-gatherer ancestry’ (WHG) which was described by other authors in Western Europe, and which was the dominant genetic component of Mesolithic populations in Western Europe, the Balkans and largely even in Northern Europe (Mathieson et al., 2018). Men from these populations carried almost exclusively Y haplogroups I-M170 and R1b-M343. So far, only one R1b-M343 sample (from Padina at the Danubian Iron Gates) was specified more precisely, as R1b-U106 (Narasimhan et al., 2018). Because the current geographical distribution of I-M170 and R1b-U106 in Central and Northern Europe is very similar, this finding supports the previously pronounced idea (cf. (Grasgruber et al., 2014)) that these Y haplogroups shared a long common history. In contrast, R1a-M420 entered the scene much later, as a marker of Eneolithic steppe cultures which were also characterized by remarkable genetic predispositions for height (Mathieson et al., 2015).

Although autosomal genetic ancestry in today’s Europe is not always perfectly in line with the distribution of Y haplogroups, both the WHG and the steppe component correlate positively with predicted genetic height, whereas Neolithic ancestry correlates negatively (Berg, Zhang & Coop, 2017). Unfortunately, except for Croats (probably from mainland Croatia) and Albanians, Berg, Zhang & Coop (2017) did not test any other modern populations from the Western Balkans. The Croats in this study had an above average predicted height in the European context, but not as tall as nations of Northern and Central Europe. In fact, their predicted stature was equal to that of Basques, French and Spaniards, which completely contradicts the fact that these nations are ∼3cm shorter than Croats, despite superior economic and nutritional statistics (see Grasgruber et al., 2014). Because the predictive power of the genetic loci (single nucleotide polymorphisms, SNPs) used in this study depends on their selection and differs from region to region, it would be necessary to conduct detailed sampling in the Dinaric Alps and identify SNPs that are specifically linked with tallness in the local population.

The problematic informative value of the currently used height markers was illustrated by a recent study that tried to challenge the magnitude of the supposed genetic differences in Europe (Sohail et al., 2018). Somewhat ironically, this study used a sample from Bosnia and Herzegovina whose predicted genetic height was entirely moderate in the European context. Apparently, if this prediction is correct, it would be necessary to find some fundamental environmental factor that could illuminate the Dinaric phenomenon. So far, our data rather support the genetic explanation.

**Figure 7:**
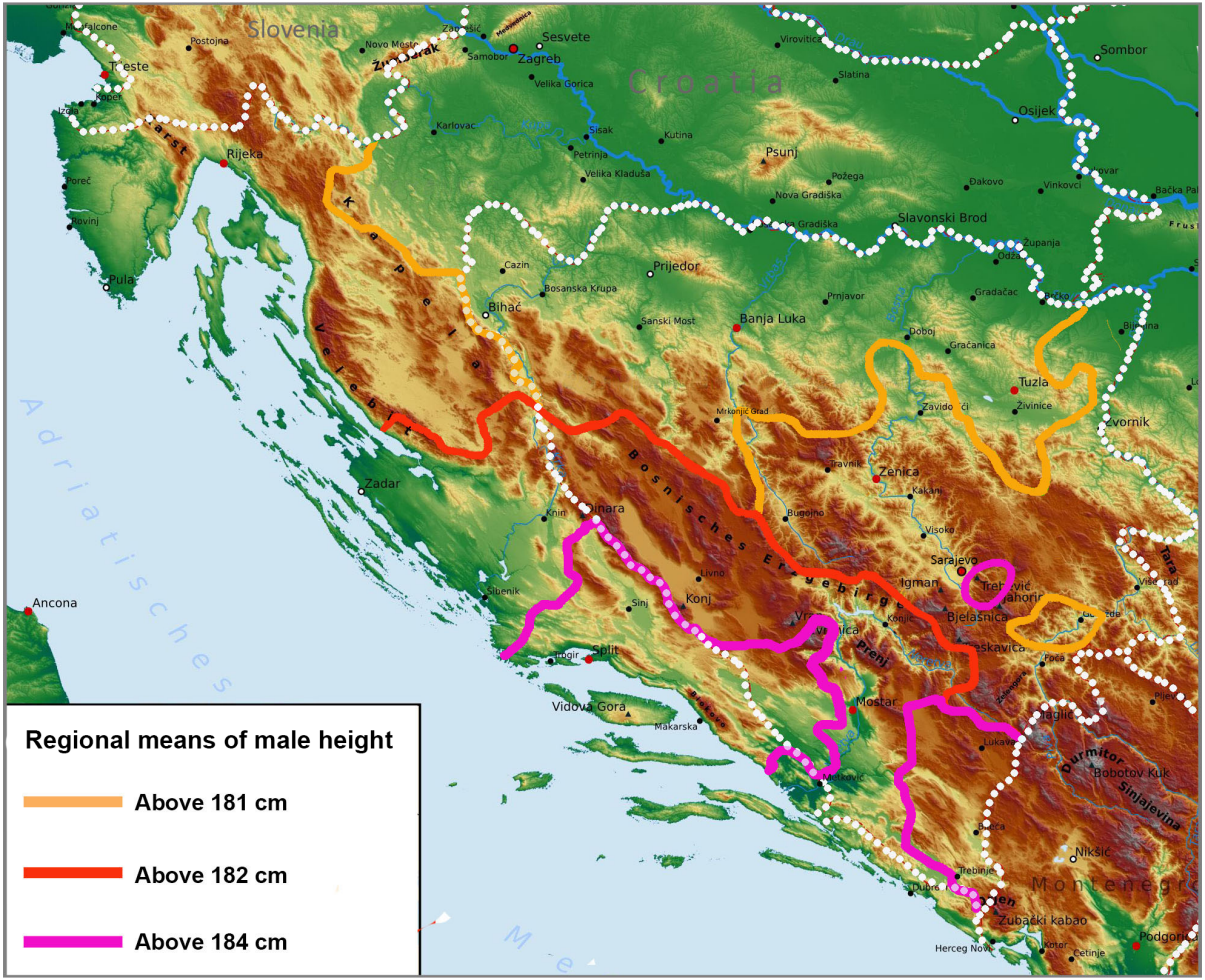
Regional means of male height on the Adriatic coast of Croatia and in Bosnia and Herzegovina (according to the self-reported place of residence) projected against the physical map of the Western Balkans.

Figure 7 shows that the area with tall male statures above 181 cm roughly copies the outer mountain ridge of the Dinaric Alps. Height gradually increases towards the core area of these mountains and peaks among the towns of Makarska–Imotski–Široki Brijeg–Čapljina–Metković, where we encounter means around 185 cm. Only the region of Bijeljina in northeastern Bosnia is an exception, possibly due to good economic conditions. Another peak may exist in central and northwestern Montenegro, in the municipalities of Kolašin & Savnik (185.5 cm, *n* = 30) and Plužine & Žabljak (184.9 cm, *n* = 28) (Popović, 2017), but the samples are small. In the same survey, the tallest Montenegrin females were found in the northwestern municipalities of Nikšić (170.9 cm, *n* = 204) and Plužine-Žabljak (170.1 cm, *n* = 34) which are values that our female sample from Dalmatia cannot match.

The taller statures in Adriatic Croatia can be, at least partly, linked with more favourable economic conditions (the benefits of tourism in coastal areas), and as we have previously shown (Grasgruber et al., 2016), height differences in Herzegovina stem from religiously conditioned dietary customs, but these factors cannot explain the exceptional height of local people in the European context, at generally below-average nutritional standards. They also do not explain why the area with exceptional statures stretches across three different countries and different types of landscape, as far as to the mountains of northwestern Montenegro. Although Coon (1970) speculated that North Albanians living on limestone are taller than their neighbours due to the high mineral content in the food chain, Buretić-Tomljanović et al. (2007) failed to find any connection between height and mean 10-year calcium concentration in drinking water in Croatia. The highest concentration of calcium was found in the central region of Croatia around Zagreb (86.1 mg/L), followed by the northwestern region (80.7 mg/L). The southern region (Dalmatia) (68.7 mg/L) and the eastern region (Slavonia) (60.9 mg/L) reached much lower values. All these data do not deviate from the usual content of calcium in European groundwater (1–100 mg/L) (Ong, Grandjean & Heaney, 2009, p. 41).

Because height in the Dinaric Alps peaks behind mountain ranges which historically served as a barrier to genetic flow, the gradient in height could be explained by the increasing proportion of admixture from continental Europe. Because we observe a 5–6 cm difference between the central and outer parts of the Dinaric Alps, it is possible that genetics could completely outweigh the role of social, economic and other environmental factors. Indeed, if we use our dataset of 56 socioeconomic, nutritional and genetic factors from seven countries of the Dinaric Alps (former Yugoslavia and Albania), the only factor with a significantly positive relationship to male height is genetic—the frequency of I-M170 (*r* = 0.84, *p* = 0.019). Infant mortality (*r* =  − 0.78, *p* = 0.039) emerges as the only meaningful variable with a negative relationship. The combined frequencies of Neolithic Y haplogroups (E-M96, G-M201 and J-M304) approach significance (*r* =  − 0.69, *p* = 0.086).

Considering that the quality of nutrition in Albania appears to be similar to that of BiH, Croatia, or Serbia, it is possible that the much higher Neolithic admixture, combined with 2–3 times higher infant mortality, could explain why the height of contemporary Albanian men is an anomaly in the Dinaric context—only 174.0 cm, which is 8.9 cm (!) shorter than in well-fed Montenegrins, and 5.5 cm shorter than in Kosovar Albanians. Preliminary results from Albanian universities confirm these large differences (S Popović, pers. comm., 2017). At the same time, the GDP per capita in Albania (for 2016) is 18% higher than in Kosovo (The World Bank). The strikingly taller statures of Kosovar men are thus surprising. At the moment, we can say that the predicted genetic height of Albanians is really markedly lower than that of Croats, but it is simultaneously also much higher than e.g., in Greeks or South Italians (Berg, Zhang & Coop, 2017).

Despite the impressive tallness documented in some Adriatic regions, it is noteworthy that our study has not demonstrated any increase of height, when compared with the study of Pineau, Delamarche & Bozinovic (2005) from the years 2001–2003. In fact, our research covered six out of the seven Dalmatian towns measured in Pineau et al.’s study (except Vrgorac), and mean male height in local schools (182.7 cm, *n* = 716) was substantially lower than in the above mentioned study (183.8 cm, *n* = 1,253). At the best case, our weighted mean of four Dalmatian counties (183.7 cm) shows that the height of Dalmatian males has not changed even after 15 years. The same applies to our female samples from schools in five Dalmatian towns (168.5 cm, *n* = 202) which lag far behind the very high values reported by Pineau et al. in Split schools (171.1 cm, *n* = 873), and are only approaching Dubrovnik schools (169.4 cm, *n* = 259). The reasons of this discrepancy are not entirely clear. Although some data in Pineau et al.’s study were obtained via personal communication and may not be perfectly reliable, it is also possible that the authors disproportionately targeted elite high schools. Either way, recent evidence shows that height in Croatia has really been stagnating or even decreasing during the last two decades.

**Figure 8:**
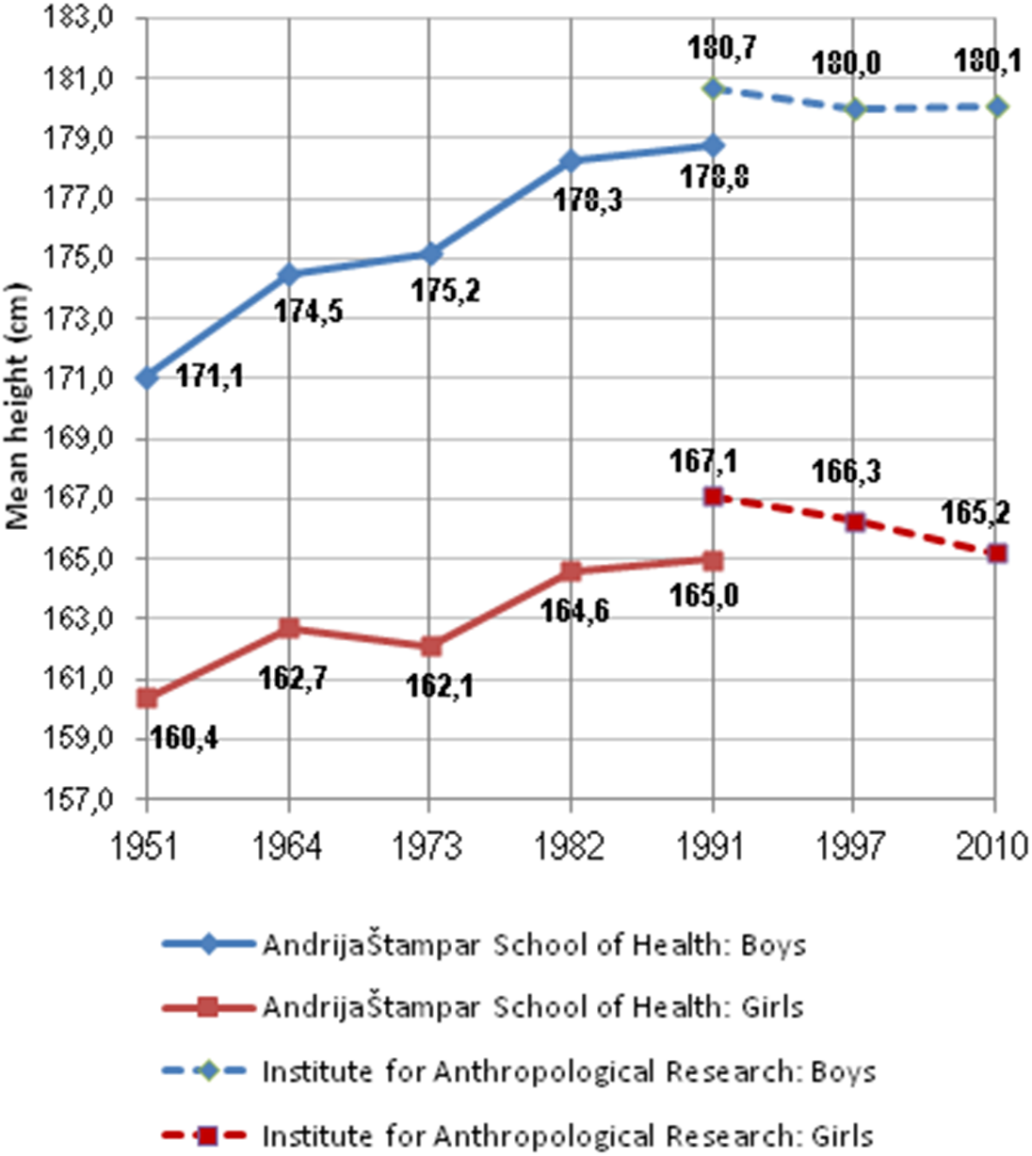
Development of the secular trend in the city of Zagreb between 1951–2010. Pooled means of 18 and 19-year olds. Based on the studies of the Andrija Štampar School of Health (Jovanović et al., 2003) and the Institute for Anthropological Research (Petranović et al., 2014).

The events of the Croatian war of independence (1991–1995) clearly influenced the growth of children in Osijek in eastern Croatia, because 7–8 year olds measured in 1995-1996 were approximately 2 cm shorter than their peers measured in 1980–1981 (Jovanović et al., 2003). In Zagreb, the research of the Andrija Štampar School of Health between 1951–1991 documented a continuous, positive height trend (Fig. 8) (Preberg, Jureša & Kujundžić, 1995). However, studies of the Institute for Anthropological Research between 1991–2010 showed a significant decrease from 180.7 cm to 180.1 cm in boys and from 167.1 cm to 165.2 cm in girls (Petranović et al., 2014). Although these two institutions apparently targeted different types of schools and the latter surveys were less representative, it is clear that no increase of height has occurred in Zagreb since 1991. This development can be connected with the dramatic decline of the economy in the early 1990s, which recovered as late as during the 2000s.

Still, if we are to believe results of nationwide surveys, the mean height of 18-year olds in Croatia has increased by 2.9 cm in boys and 1.8 cm in girls between 1980–84 and 2006–08 (Jureša, Musil & Kujundžić Tiljak, 2012). Historical data show that between 1883–2008 (125 years), the average Croat male has grown by 15 cm (from 165.5 cm to 180.5 cm) which means a mean rate of 1.2 cm/decade. Based on the information listed by Coon (1939, p. 591), we can estimate that the height gain of young men in the county of Istra has been approximately 15 cm as well. According to the records of Austrian–Hungarian recruits around the year 1870 (Komlos, 2007), young men from the town of Zadar (Zara) were the tallest in the whole empire at 168.7 cm, which is a difference of +14.1 cm, when compared to the present study (1.0 cm/decade). However, it should be noted that these historical data do not necessarily represent the same populations, due to migrations to the coastal regions occurring in the course of the 20th century (S Popović, pers. comm., 2018).

Historical information from other regions of the Adriatic area is scarcer. As already mentioned, the most recent nationwide survey comparable with the dimension of our work was conducted in 1980–1984 (Prebeg, 1988). Another nationwide survey was performed in 1991–1993, but was limited to a mere four areas (Prebeg, 1998). Few detailed numbers from these studies were published, but some rough data on Split are available (Jovanović et al., 2003). They show that in 1980–1984, the mean height of 18-year olds in Split was 181 cm in boys and 167 cm in girls. In 1992, it reached 182.5 cm and 170 cm, respectively, although the height of girls from this latter survey appears implausibly high. Provided that Pineau, Delamarche & Bozinovic’s (2005) result from Split (184.5 cm in boys, 171.1 cm in girls) is not far from reality, the secular trend in Dalmatia must have continued during the subsequent economic depression. On the other hand, our mean of Croatian boys from Split schools is again the same as in 1992 (182.5 cm, *n* = 227). This would mean that economic disturbances in the country have affected even Dalmatia since 2001–2003. In any case, the available data show that current male height in Adriatic Croatia is not higher than in the early 1990s.

## Conclusion

In summary, the data presented in our article demonstrate that people from Dalmatia currently belong to the tallest in the world, and local young men are even the tallest in the age category of 18 years. However, this phenomenon is limited only to the territory of Dalmatia, Herzegovina and Montenegro. Other regions of Croatia are characterized by a much shorter stature and these large regional differences have persisted at least since the end of the 19th century. Because we are not aware of any environmental factor that could be responsible for these geographical trends, specific genetic predispositions shared by these populations are the most likely explanation. In the near future, the mutual relationship between genetic and environmental factors will hopefully be illuminated in detail, but such a research project would require targeting the whole core area of the Dinaric Alps.

Another fundamental finding is the fact that the secular height trend in Croatia has been negatively influenced by the economic depression in the 1990s. The onset of the economic crisis in 2008 further delayed a marked improvement in living standards. However, because the quality of nutrition in Croatia is still below the European standards, a further continuation of the positive height trend is possible. These conclusions can have very important practical implications because height is a key factor in sports success (Ackland, Elliott & Bloomfield, 2009). Information on body proportions (relatively long legs and rather short arm span) can further help in identifying sports that would be most suitable for the Dinaric body type. A more intensive focus of the local governments on the nutritional and health status of the young generation should subsequently pay off richly in the form of international sports prestige.

##  Supplemental Information

10.7717/peerj.6598/supp-1 Supplemental Information 1 Supplemental tables and figures

10.7717/peerj.6598/supp-2 Supplemental Information 2 Supplemental dataset

10.7717/peerj.6598/supp-3 Supplemental Information 3 Questionnaire

## Additional Information and Declarations

### Data Availability

The following information was supplied regarding data availability: The raw data are available in the Supplementary Dataset.
