# Supplementary material for: The coast of giants: an anthropometric survey of high schoolers on the Adriatic coast of Croatia
Source: PeerJ. 2019 Apr 17;7:e6598. doi: 10.7717/peerj.6598 (PMC6475134; doi:10.7717/peerj.6598)
Supplement: Supplemental Information 1 [file peerj-07-6598-s001.pdf]

Supplemental tables and figures

Supplemental Table S1. Proportion of various types of schools in the measured samples, by county.

County *(županija)*	*Available schools*				*Measured schools*			
	Gimnazije[1]	**'Mixed high schools'**[2]	**Other schols**	***TOTAL***	***Gimnazije***[1]	**'Mixed high schools'**[2]	**Other schols**	***TOTAL (% of all schools)***
Split-Dalmacija	*8*	*9*	*27*	*44*	*2*	*2*	*13*	*17 (38.6%)*
Dubrovnik-Neretva	*4*	*5*	*7*	*16*	*2*	*2*	*3*	*7 (43.8%)*
Šibenik-Knin	*2*	*2*	*9*	*13*	*1*	*2*	*7*	*10 (76.9%)*
Zadar	*5*	*5*	*12*	*22*	*1*	*2*	*3*	*6 (27.3%)*
Primorje-Gorski Kotar	*5*	*8*	*20*	*33*	*2*	*0*	*4*	*6 (18.2%)*
Istra	*4*	*9*	*13*	*26*	*2*	*1*	*5*	*8 (30.8%)*
Lika-Senj	*1*	*3*	*1*	*5*	*0*	*3*	*1*	*4 (80.0%)*
Karlovac	*3*	*2*	*9*	*14*	*2*	*0*	*7*	*9 (64.3%)*
**TOTAL**	***32***	***43***	***98***	***173***	***12***	***12***	***43***	***67 (38.7%)***

Notes: [1]*Gimnazije* = elite high schools. [2] 'Mixed high schools' *(Srednje škole)* = schools combining several different classes, usually *gimnazije* and economic schools.

Supplemental Table S2. Means of the measured schools. The data include both domestic high schoolers (Supplemental dataset, Sheets 1-2) and high schoolers coming from other regions (Supplemental dataset, Sheet 3-4).

**County** ***(županija)*** TOWN, school	Males				Females			
	n	Age in years (SD)	Height (cm) Mean (SD)	Height (cm) Maximum Minimum	*n*	Age in years (SD)	Height (cm) Mean (SD)	Height (cm) Maximum Minimum
**Split-Dalmacija**	***449***	**18.2±0.4**	**184.0±6.9**	**165.0-204.5**	***155***	**18.5±0.5**	**169.0±5.8**	**151.5-186.0**
IMOTSKI, Gimnazija & Ekonomska[1]	*24*	18.5±0.3	185.7±6.8	171.2-201.8	*28*	18.7±0.3	170.6±5.8	151.5-179.6
SPLIT, Graditeljsko-geodetska tehnička	*34*	18.4±0.5	185.5±5.8	174.4-200.4				
SINJ, Tehnička i industrijska	*63*	18.4±0.3	185.1±7.4	165.0-204.5	*8*	18.1±0.2	167.0±4.8	158.2-174.0
MAKARSKA, Srednja strukovna	*18*	18.2±0.5	184.8±6.1	174.2-200.7	*23*	18.3±0.6	169.4±5.1	162.4-181.5
TROGIR, SŠ Ivana Lučića	*29*	18.1±0.3	184.4±6.1	168.6-196.6				
MAKARSKA, Srednja škola	*28*	18.6±0.3	184.4±7.0	169.0-200.6				
IMOTSKI, Tehnička	*21*	18.6±0.4	184.2±7.9	165.8-204.5				
SPLIT, Ekonomsko-birotehnička	*61*	18.3±0.5	184.2±7.6	166.3-202.4				
SPLIT, Prva gimnazija	*41*	18.1±0.3	184.0±7.2	168.5-203.8				
SPLIT, Zdravstvena	*10*	18.8±0.5	183.5±4.9	175.1-183.5	*41*	18.7±0.6	169.4±5.8	155.0-180.7
SINJ, Strukovna bana J. Jelačića	*22*	18.3±0.4	183.1±6.1	173.9-196.0	*49*	18.4±0.3	168.0±6.1	158.6-186.0
IMOTSKI, Obrtničko-industrijska	*16*	17.9±0.2	182.6±4.4	172.8-190.5	*6*	17.6±0.2	167.1±2.9	163.5-171.3
SPLIT, Tehnička škola za strojarstvo	*14*	17.9±0.3	182.4±6.3	171.9-192.7				
SPLIT, Pomorska	*40*	17.9±0.2	182.1±6.7	167.0-192.5				
SPLIT, Škola za dizajn, grafiku	*19*	17.9±0.4	182.0±7.3	168.8-196.5				
SPLIT, Likovnih umjetnosti	*9*	18.3±0.4	181.1±5.5	168.3-187.0				
**Dubrovnik-Neretva**	***242***	**18.4±0.4**	**183.7±6.7**	**166.6-201.8**	***39***	**18.4±0.2**	**169.1±7.1**	**156.2-188.0**
METKOVIĆ, Gimnazija	*29*	18.1±0.3	185.2±7.7	169.9-197.7	*39*	18.4±0.2	169.1±7.1	156.2-188.0
DUBROVNIK, Pomorsko-tehnička	*88*	18.6±0.4	184.3±6.5	170.0-201.8				
METKOVIĆ, Srednja škola	*30*	18.0±0.2	183.7±5.8	172.4-196.8				
DUBROVNIK, Turistička i ugostiteljska	*21*	18.5±0.3	183.3±5.0	175.0-193.9				
DUBROVNIK, Ekonomska i trgovačka	*16*	18.7±0.5	183.2±7.2	172.5-198.1				
DUBROVNIK, Gimnazija	*47*	18.6±0.3	182.4±6.9	166.6-197.8				
PLOČE, SŠ fra Andrije Kačića Miošića	*11*	18.0±0.3	181.7±7.2	175.1-196.2				
**Šibenik-Knin**	***209***	**18.5±0.4**	**183.4±6.6**	**163.5-205.5**	***74***	**18.9±0.6**	**167.9±5.1**	**156.0-179.2**
KNIN, Srednja škola Lovre Montija	*14*	18.6±0.4	185.0±5.9	176.8-195.7				
DRNIŠ, SŠ I. Mestrovića	*42*	18.4±0.3	184.4±7.2	167.2-203.4				
ŠIBENIK, Ekonomska	*30*	18.3±0.4	184.4±5.2	175.0-195.6				
ŠIBENIK, Gimnazija A. Vrančića	*26*	18.4±0.3	184.1±5.7	167.1-193.5				
ŠIBENIK, Prometno-tehnička	*34*	18.5±0.4	184.0±7.7	169.8-205.5				
ŠIBENIK, Turističko-ugostiteljska	*19*	18.4±0.4	182.5±5.9	172.0-192.5	*23*	18.3±0.3	167.9±5.3	158.5-179.2
ŠIBENIK, Medicinska	*13*	19.3±0.4	182.0±5.5	171.5-189.0	*43*	19.2±0.4	167.7±5.2	156.0-178.9
KNIN, Sr. struk. škola Kralja Zvonimira	*25*	18.5±0.3	181.6±5.8	169.0-195.4				
ŠIBENIK, Srednja strukovna	*1*	18.2	178.6		*8*	19.0±0.7	168.8±4.0	161.8-174.3
ŠIBENIK, Industrijsko-obrtnička	*5*	18.4±0.2	175.7±7.3	163.5-184.4				
**Zadar**	***251***	**18.4±0.4**	**182.7±6.1**	**167.1-200.7**	***19***	**18.3±0.5**	**168.1±6.8**	**151.4-183.0**
ZADAR, Gimnazija J. Barakovića	*39*	18.4±0.3	184.6±5.6	173.8-195.0				
BENKOVAC, Sr. škola kneza Branimira	*30*	18.5±0.3	184.0±7.1	167.1-200.7				
ZADAR, Pomorska	*57*	18.5±0.5	182.5±5.1	170.9-190.9				
ZADAR, Strukovna V. Vlatkovića	*50*	18.5±0.4	182.4±6.3	168.6-197.0				
ZADAR, Tehnička	*62*	18.4±0.3	182.0±6.1	171.5-198.0				
GRAČAC, Srednja škola	*13*	18.0±0.6	178.8±5.9	172.0-192.0	*19*	18.3±0.5	168.1±6.8	151.4-183.0

[1] Gimnazija and Ekonomska škola Imotski are located in the same building and their results are merged together.

County *(županija)* TOWN, school	Males				Females			
	n	Age in years (SD)	Height (cm) Mean (SD)	Height (cm) Maximum Minimum	*n*	Age in years (SD)	Height (cm) Mean (SD)	Height (cm) Maximum Minimum
**Primorje-Gorski Kotar**	***243***	**18.7±0.4**	**181.9±6.7**	**163.0-199.3**				
RIJEKA, Prometna	*19*	18.8±0.5	183.5±7.5	172.7-198.4				
RIJEKA, Prva riječka hrvat. gimnazija	*60*	18.7±0.5	183.3±6.6	166.6-199.3				
RIJEKA, Gradevinska tehnička	*35*	18.8±0.6	182.0±6.9	166.9-194.4				
RIJEKA, Prva sušacka hrvat. gimnazija	*27*	18.6±0.3	181.5±6.6	169.3-193.3				
RIJEKA, Prirodoslovna i grafička	*43*	18.7±0.4	181.4±6.4	167.8-198.0				
BAKAR, Pomorska	*59*	18.7±0.4	180.4±6.3	163.0-191.5				
**Lika-Senj**	***82***	**18.5±0.4**	**181.2±7.0**	**165.5-196.7**	***49***	**18.5±0.4**	**167.6±5.8**	**152.3-180.2**
KORENICA, Sr. škola Plitvička jezera	*11*	18.5±0.4	183.5±4.4	174.5-191.0	*16*	18.6±0.3	167.9±6.3	153.6-180.2
SENJ, Srednja škola P. Rittera	*15*	18.6±0.3	183.0±5.2	172.2-191.4				
OTOČAC, Srednja škola	*19*	18.6±0.5	181.3±8.0	166.5-196.7	*22*	18.5±0.4	167.0±5.4	152.3-177.1
GOSPIĆ, Strukovna	*37*	18.5±0.3	179.7±7.3	165.5-192.9	*11*	18.5±0.3	168.4±5.9	160.0-179.0
**Istra**	***139***	**18.6±0.4**	**181.1±6.6**	**166.4-203.5**	***225***	**18.5±0.5**	**166.7±6.7**	**148.2-182.6**
PULA, Tehnička	*13*	18.4±0.3	183.0±4.9	171.7-190.5	*5*	18.5±0.3	158.8±3.1	152.9-162.2
PULA, Primijenjenih umjetnosti,dizajna	*4*	18.4	182.9	175.0-187.8	*21*	18.0±0.7	164.1±4.5	152.8-171.2
PULA, Ekonomska	*22*	18.5±0.3	182.3±7.1	171.4-194.5	*53*	18.5±0.4	167.2±6.5	151.6-181.0
PULA, Priv. gimnazija "Juraj Dobrila"	*3*	18.5	182.0	175.1-194.3				
PULA, Strukovna	*2*	19.7	181.4	179.2-183.5	*24*	18.7±0.5	168.8±8.1	148.2-178.9
PULA, Medicinska	*18*	18.9±0.4	180.6±4.7	166.4-187.7	*30*	19.0±0.7	167.0±6.9	150.3-182.6
PULA, Gimnazija	*67*	18.5±0.3	180.5±6.9	167.8-203.5	*92*	18.5±0.3	166.8±6.3	153.3-182.4
PULA, Talijanska srednja	*10*	18.4±0.3	180.1±7.2	167.9-190.4				
**Karlovac**	***212***	**18.6±0.5**	**180.4±6.2**	**162.0-195.3**	***231***	**18.6±0.4**	**166.6±6.0**	**151.2-181.3**
KARLOVAC, Prirodoslovna	*8*	18.3±0.3	185.3±6.6	175.0-195.3	*27*	18.6±0.4	167.3±5.3	157.8-176.0
KARLOVAC, Ekonomsko-turistička	*23*	18.6±0.3	181.7±7.0	162.6-193.2	*64*	18.6±0.4	165.3±6.0	155.0-180.9
KARLOVAC, Trgovačko-ugostiteljska	*4*	18.9	181.5	176.6-186.7	*16*	18.8±0.6	167.3±7.2	155.0-181.3
KARLOVAC, Gimnazija	*40*	18.5±0.4	180.9±6.7	163.8-194.2	*56*	18.5±0.3	168.1±6.1	153.4-180.6
OGULIN, Obrtnička i tehnička	*41*	18.4±0.3	180.4±6.2	167.8-195.2				
KARLOVAC, Medicinska	*27*	18.9±0.5	180.1±4.8	172.2-190.6	*38*	18.5±0.4	164.7±5.0	151.2-173.3
KARLOVAC, Tehnička	*42*	18.8±0.5	179.8±5.7	168.6-190.5	*1*	18.6	166.9	
KARLOVAC, Šumarska i drvodjeljska	*21*	18.7±0.6	178.7±5.6	162.0-190.3	*4*	18.7	166.1	161.6-170.1
OGULIN, Gimnazija B. Frankopana	*6*	18.8±0.3	175.6±3.4	171.1-180.7	*25*	18.4±0.3	168.1±6.0	155.4-178.5
**TOTAL**	***1827***	**18.5±0.5**	**182.7±6.7**	**162.0-205.5**	***792***	**18.6±0.5**	**167.4±6.2**	**148.2-188.0**

Suplemental Table S3. Population size in the targeted counties and mean height in the whole area, corrected for population size in counties.

	Population	% of total	Mean male height (cm)	Mean female height (cm)
Istra	208,055	13.5	181.1	166.7
Karlovac	128,899	8.4	180.6	166.6
Primorje-Gorski Kotar	296,195	19.2	181.9	-
Lika-Senj	50,927	3.3	181.0	168.0
Zadar	170,017	11.0	182.8	167.5
Šibenik-Knin	109,375	7.1	183.4	167.9
Split-Dalmacija	454,798	29.5	184.1	168.9
Dubrovnik-Neretva	122,568	8.0	183.6	169.1
**TOTAL**	**1,540,834**	**100.0**	**183.6**	**168.0***

Note: *The female mean does not include the county of Primorje-Gorski Kotar, from which only five girls were measured. After the inclusion of this county, the mean female height would marginally increase to 168.2 cm.

Supplemental Table S4. Proportion of parents with university education. Comparison of data reported by high schoolers with the official data from the Croatian Bureau of Statistics for 2011 (www.dzs.hr).

County (*županija*)	***DATA REPORTED BY HIGH SCHOOLERS***				***DATA BY www.dzs.hr (for 2011)***			
	Males		***Females***		***% of adults with higher education in the county***			
					All forms[1]		***University only***	
	n	***% univ. educated parents***	***n***	***% univ. educated parents***	***35-39 years***	***40-44 years***	***35-44 years***	***40-44 years***
Split-Dalmacija	*359**	*27.7*	*54**	*23.1*	*22.9*	*18.9*	*14.8*	*11.2*
Dubrovnik-Neretva	*241*	*34.0*	*38**	*30.3*	*26.8*	*20.1*	*15.6*	*11.4*
Šibenik-Knin	*193**	*21.5*	*23**	*34.8*	*18.8*	*14.5*	*11.3*	*7.7*
Zadar	*249*	*18.9*	*20*	*0.0*	*21.6*	*15.8*	*14.5*	*10.0*
**Four Dalmatian counties**	***1042***	***27.3***	***135***	***23.7***	***22.7***	***17.9***	***14.4***	***11.0***
Primorje-Gorski Kotar	*238*	*29.6*	*5*	*50.0*	*27.0*	*22.9*	*18.2*	*14.4*
Istra	*143*	*32.9*	*225*	*30.0*	*22.7*	*18.4*	*14.4*	*10.3*
Lika-Senj	*86*	*16.3*	*48*	*12.5*	*15.0*	*11.9*	*8.1*	*6.1*
Karlovac	*199*	*25.1*	*221**	*21.0*	*17.4*	*14.6*	*10.3*	*8.0*
**Four northern counties**	***666***	***25.9***	***499***	***24.5***	***23.1***	***19.2***	***14.9***	***11.3***
**TOTAL**	***1708***	***26.4***	***634***	***24.4***	***22.9***	***18.5***	***14.6***	***11.1***

Note: *Information on the education of parents was not available for all high schoolers in these counties. [1]Includes all forms of education higher than high school.

Supplemental Table S5. Correlations between male and female height, and socioeconomic variables in eight counties. (See Supplemental Figures S1-S4 below)

**County *(županija)***	**Male height (cm) in 8 counties**	**Female height (cm) in 8 counties**
Net salary 1995-2000	0.36 (p=0.39)	0.19 (p=0.65)
Net salary 2000-2014	0.02 (p=0.97)	0.15 (p=0.72)
**Net salary 1995-2014**	**0.07 (p=0.87)**	**0.15 (p=0.73)**
Unemployment 1998-2000	0.41 (p=0.31)	-0.04 (p=0.93)
Unemployment 2000-2014	0.32 (p=0.45)	0.04 (p=0.93)
**Unemployment 1998-2014**	**0.33 (p=0.42)**	**0.03 (p=0.95)**

Source: Croatian Bureau of Statistics, www.dzs.hr

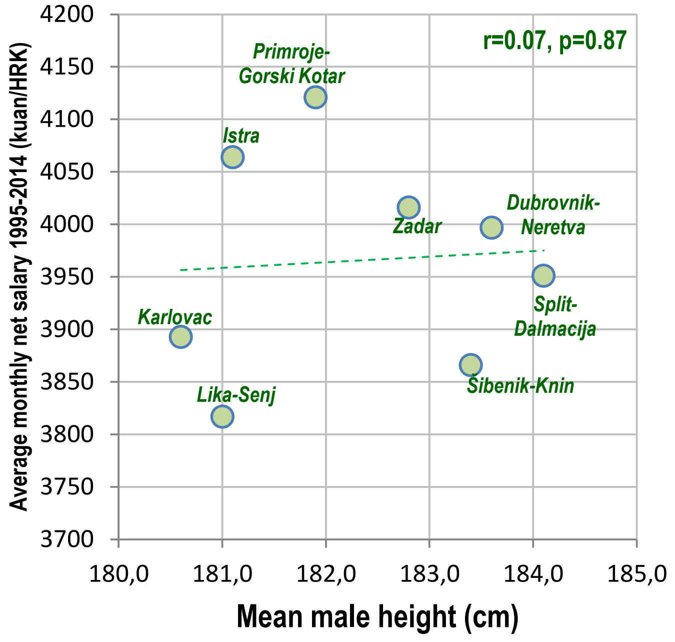

Supplemental Figure S1. Mean male height in eight counties and mean monthly net salary between 1995-2014.

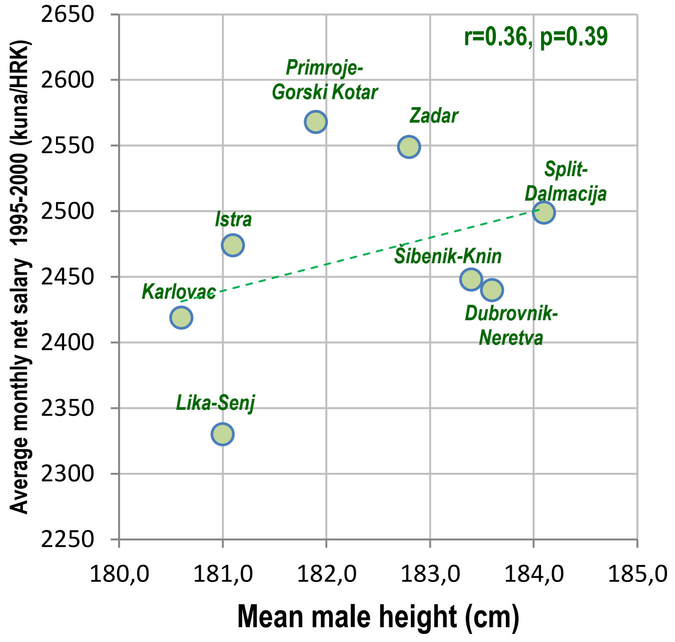

Supplemental Figure S2. Mean male height in eight counties and mean monthly net salary between 1995-2000.

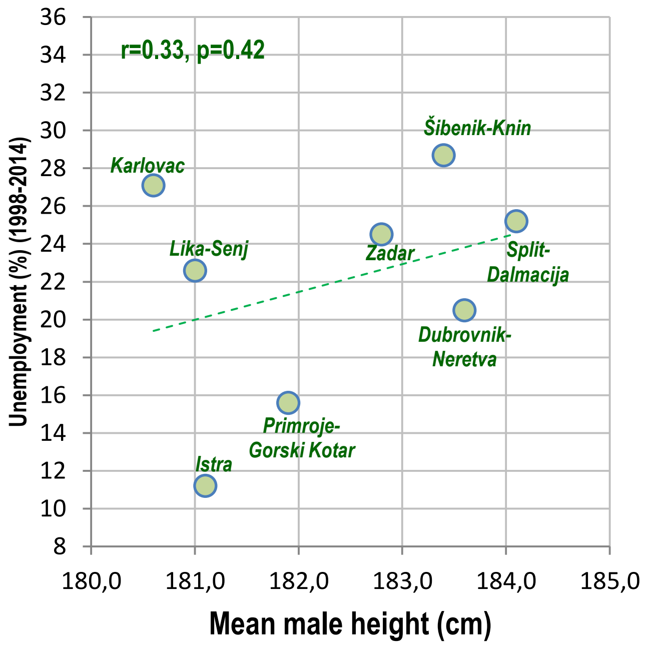

Supplemental Figure S3. Mean male height in eight counties and unemployment between 1998-2014.

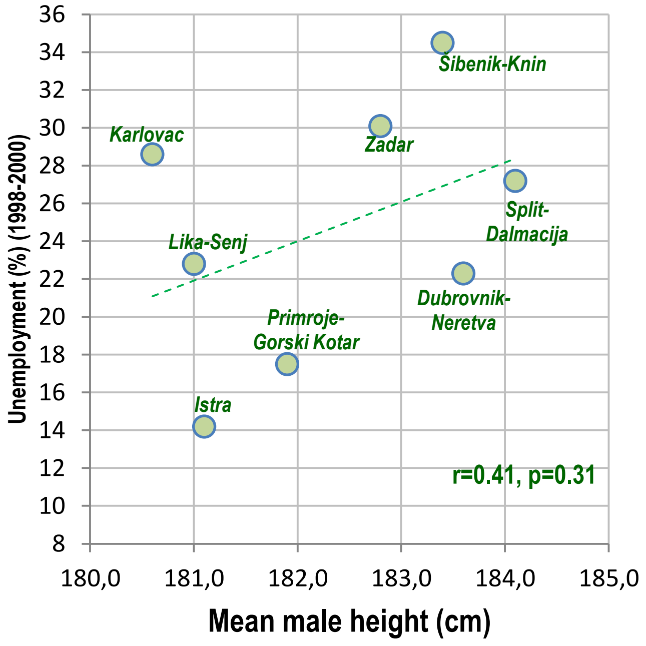

Supplemental Figure S4. Mean male height in eight counties and unemployment between 1998-2000.

Supplemental Table S6. Multiple regression models of male height and two socioeconomic variables in eight counties, by different time periods.

MODELS	(1)	(2)	(3)
Net salary 1995-2000	b* = 0.47<br>r = 0.49<br>p = 0.27		
Net salary 2000-2014		b* = 0.81<br>r = 0.50<br>p = 0.26	
**Net salary 1995-2014**			**b* = 0.67<br>r = 0.48<br>p = 0.27**
Unemployment 1998-2000	b* = 0.46<br>r = 0.47<br>p = 0.28		
Unemployment 2000-2014		b* = 0.97<br>r = 0.57<br>p = 0.18	
**Unemployment 1998-2014**			**b* = 0.82<br>r = 0.56<br>p = 0.19**
**Adj. R^2**	**0.051**	**0.053**	**0.047**
**p-value**	***p* = 0.37**	***p* = 0.38**	***p* = 0.38**

*Note: b** = *beta* coefficient (displays how many standard deviations a dependent variable will change, per 1 standard deviation increase in the independent variable); *r* = partial correlation in the model; *p* = probability value.
