# Supplementary material for: The coast of giants: an anthropometric survey of high schoolers on the Adriatic coast of Croatia
Source: PeerJ. 2019 Apr 17;7:e6598. doi: 10.7717/peerj.6598 (PMC6475134; doi:10.7717/peerj.6598)
Supplement: Supplemental Information 3 [file peerj-07-6598-s003.pdf]

Antropološka istraživanja sportskog potencijala na hrvatskoj obali Jadrana

PhDr. Jan Cacek, Ph.D., Bc. Pavel Grasgruber, Mgr. Eduard Hrazdíra, Ph.D., Bc. Nikola Stračárová, Mgr. Tereza Králová, Mgr. Tereza Hammerová, Mgr. Sylva Hřebíčková, Ph.D.
Fakulta sportovních studií MU, Kamenice 753/5, Univerzitní kampus Bohunice, budova A34,
625 00 Brno

Informirani pristanak

Ja, dolje potpisani, se slobodno i dobrovoljno slažem s mojim sudjelovanjem u istraživanju „Antropološka istraživanja sportskog potencijala na hrvatskoj obali Jadrana“, u okviru kojeg ću sudjelovati u antropološkom istraživanju visine, sjedišne visine i raspona ruku. Također se slažem s unosom podataka o mjestu stanovanja, obrazovanju roditelja i datumu rođenja.

Dobio sam objašnjenje od jednog od sudionika projekta (PhDr. Jan Cacek, Ph.D., Bc. Pavel Grasgruber, Mgr. Eduard Hrazdíra, Ph.D., Bc. Nikola Stračárová, Mgr. Tereza Králová, Mgr. Tereza Hammerová, Mgr. Sylva Hřebíčková, Ph.D.) o prirodi i razlozima studije. Rečeno mi je sve što se od mene očekuje. Imao sam se priliku raspitati o svim otvorenim pitanjima (gore spomenuta) i potpuno razumjeti sve informacije koje sam dobio. Ja sam svjestan da se studija može prekinuti u bilo kojem trenutku. Svjestan sam činjenice da sudjelovanje u istraživanju za mene nema druge prednosti ili pogodnosti.

Ja znam da će moji rezultati biti u okviru znanstvenog vrednovanja provedeni pod šifrom, zajedno s podacima o godini rođenja. Bez mog daljnjeg pisanog informiranog pristanka rezultati se ne mogu se koristiti za druga istraživanja. Istovremeno dajem pristanak za obradu mojih osobnih podataka potrebnih za dovršetak istražnog procesa istraživanja. Bio sam upoznat s činjenicom da će ovi podaci biti izbrisani u trenutku završetka mog sudjelovanja u istraživanju ili preko moje osobne ili pisane obavijesti o raskidu sudjelovanja ili automatski nakon pola godine od mog zadnjeg posjeta.

Dajem suglasnost na gore navedene uvjete provedbe istraživanja.

MOLIMO ISPUNITE:

□ muško□ žensko

Datum rođenja (dan, mjesec, godina)……………………..............…

Naziv škole...

Mjesto stanovanja……………………......……...Županija………………………………….

Imaju vaši roditelji univerzitetsko obrazovanje? (sveučilište)□ otac □ majka

Hvala vam na suradnji. **Potpis ………………........…………….…**

TĚLESNÁ VÝŠKA……………………........…..

VÝŠKA V SEDĚ……………………........……

ROZPĚTÍ PAŽÍ……………………........……...
